# Dissociating Frontal Lobe Lesion Induced Deficits in Rule Value Learning Using Reinforcement Learning Models and a WCST Analog

**DOI:** 10.1523/ENEURO.0117-25.2025

**Published:** 2025-05-16

**Authors:** Lucie Capkova, Matthew Ainsworth, Farshad A. Mansouri, Mark J. Buckley

**Affiliations:** ^1^Department of Experimental Psychology, University of Oxford, Oxford OX1 3SR, United Kingdom; ^2^Cognitive Neuroscience Laboratory, Department of Physiology, Monash Biomedicine Discovery Institute, Monash University, Melbourne, Victoria 3800, Australia; ^3^ARC Centre of Excellence for Integrative Brain Function, Monash University, Melbourne, Victoria 3800, Australia

**Keywords:** cingulate cortex, decision-making, prefrontal cortex, reinforcement learning, WCST

## Abstract

Distinct frontal regions make dissociable contributions to rule-guided decision-making, including the ability to learn and exploit associations between abstract rules and reward value, maintain those rules in memory, and evaluate choice outcomes. Value-based learning can be quantified using reinforcement learning (RL) models predicting optimal trial-wise choices and estimating learning rates, which can then be related to the intact functioning of specific brain areas by combining a modeling approach with lesion-behavioral data. We applied a three-parameter feedback-dependent RL model to behavioral data obtained from macaques with circumscribed lesions to the principal sulcus (PS), anterior cingulate cortex (ACC), orbitofrontal cortex (OFC), superior dorsolateral prefrontal cortex (sdlPFC), and frontopolar cortex (FPC) performing a Wisconsin card sorting task (WCST) analog. Our modeling-based approach identified distinct lesion effects on component cognitive mechanisms contributing to WCST performance. OFC lesions decreased the rate of rule value updating following both positive and negative feedback. In contrast, we found no deficit in rule value updating following PS lesions, which instead made monkeys less likely to repeat correct choices when rule values were well established, suggesting a crucial role of the PS in the working memory maintenance of rule representations. Finally, ACC lesions produced a specific deficit in learning from negative feedback, as well as impaired the ability to repeat choices following highly surprising reward, supporting a proposed role for ACC in flexibly switching between a trial-and-error mode and a working memory mode in response to increased error likelihood.

## Significance Statement

Successful rule-based decision-making in the Wisconsin card sorting task (WCST) requires control over multiple cognitive functions. To identify key latent processes that contribute to WCST performance and relate them to the intact functioning of distinct frontal lobe areas, we combined macaque lesion-behavioral data with reinforcement learning modeling. Orbitofrontal cortex lesions impaired rule value learning from both positive and negative feedback. Principal sulcus lesion-related deficits were consistent with impoverished maintenance of rule representations in working memory. Impairments in the anterior cingulate cortex lesion group likely reflected the inability to flexibly switch between trial-and-error and working memory response modes. Our results therefore highlight a modeling-based approach to distinguishing between a rule value updating and working memory-based component of the WCST.

## Introduction

Successful rule-based decision-making in volatile environments often requires control over multiple cognitive processes. Selecting optimal behavioral strategies can depend on the ability to identify rules, maintain rules in memory, evaluate decision outcomes, and generalize to novel exemplars. One popular neuropsychological assessment tool for investigating abstract rule-based decision-making in patients with dysfunctional prefrontal cortex is the Wisconsin card sorting task (WCST; Milner, 1963; Drewe, 1974; Nelson, 1976). Successful performance in this task relies on multiple cognitive processes engaging a network of interconnected brain regions. Computerized analogs of the WCST have also been used extensively in animal models (Dias et al., 1997; Mansouri and Tanaka, 2002; Nakahara et al., 2002; Moore et al., 2009; Sleezer et al., 2016; Goudar et al., 2024), facilitating detailed analyses and more targeted interventions and recordings than possible in humans (for reviews, see Nakahara et al., 2007; Mansouri et al., 2009, 2017a, 2020b).

One particular analog of the WCST used extensively in macaque lesion-behavioral studies (Mansouri et al., 2007, 2014, 2015, 2020a, 2022, 2024; Buckley et al., 2009; Kuwabara et al., 2014) and macaque electrophysiological studies (Mansouri et al., 2006, 2007, 2014; Kuwabara et al., 2014) has helped determine the mechanistic underpinnings and relative contributions of different primate cortical regions to abstract rule-guided decision-making. In this particular WCST analog, animals are presented with a central visual sample stimulus which varies from trial to trial and to obtain reward in any given trial must select which one of three surrounding visual stimuli matches it based on one of two abstract rules (match-by-color and match-by-shape). Importantly, the currently reinforced rule is never cued and must be inferred from choice feedback. Moreover, periodically the rule changes, also unannounced, and so subjects must redetermine which rule is reinforced, multiple times per session. The same WCST analog has been used in human studies, showing converging evidence of functional specialization in rule-guided decision-making in humans and macaques (Mansouri et al., 2016, 2020a; Boschin et al., 2017a,b; Fehring et al., 2022).

Successful performance in this multifaceted task depends on the orchestrated contributions of multiple cognitive abilities. Previous circumscribed lesion studies in macaques have revealed that lesions to four distinct frontal cortical regions, namely, principal sulcus (PS) within the dorsolateral prefrontal cortex, orbitofrontal cortex (OFC) on the ventral aspect of prefrontal cortex, frontopolar cortex (FPC) situated at the most anterior aspect of prefrontal cortex, and anterior cingulate cortex (ACC) in the medial frontal lobe, all have different effects on WCST performance (Mansouri et al., 2007, 2014, 2015; Buckley et al., 2009; Kuwabara et al., 2014). Across trials, abstract rules must be maintained in working memory and only PS lesions impaired this aspect of task performance (Buckley et al., 2009). Optimum task performance depends upon engaging cognitive control and only ACC lesions impaired the normal tendency to slow down under uncertainty (Buckley et al., 2009; Tanaka et al., 2013). Only one frontal lesion (sdlFC) had no effect on WCST (Buckley et al., 2009) but did impair metacognition in a different task (Kwok et al., 2019). Notably, only OFC lesions impaired the rapid relearning of rule value, which is necessary to maximize reward as the task cannot be solved by learning about individual stimulus features (colors, shapes, and locations).

That OFC is implicated in representing abstract rule value in WCST is consistent with a body of literature linking OFC with reward valuation and learning through positive reinforcement (Rudebeck et al., 2008; Rudebeck and Murray, 2008; Chau et al., 2015). Such value-based learning may be quantified by reinforcement learning (RL) models which predict future actions based on maximizing reward. RL models assume agents use feedback information to update action–reward associations and that behavior is biased toward performing actions that maximize expected rewards (Sutton and Barto, 1998). RL models previously applied to human WCST data showed that such computational modeling can identify latent cognitive mechanisms contributing to performance (Steinke et al., 2020). Crucially, RL models can provide numerical estimates of the learning rates of actors, as well as trial-by-trial predictions of choice values, which can be related to the intact functioning of distinct brain regions (Costa et al., 2016).

Here to investigate the involvement of frontal regions generally, and OFC specifically, in evaluating feedback information for learning rule value in WCST, we combine lesion-behavioral data with RL modeling. We applied a feedback-dependent RL model (FD-RL) capturing both positive and negative learning independently, and by assessing the effect of distinct lesions on model parameter values, we investigated the contribution of the ACC, PS, OFC, sdlPFC, and FPC to different aspects of WCST performance. We also related performance to trial-by-trial value of the reward prediction error, to further investigate how prefrontal lesions affect choice behavior when a reinforced rule is yet to be identified via trial-and-error versus when the abstract rule values are established.

## Materials and Methods

### Task design

**Figure 1.**
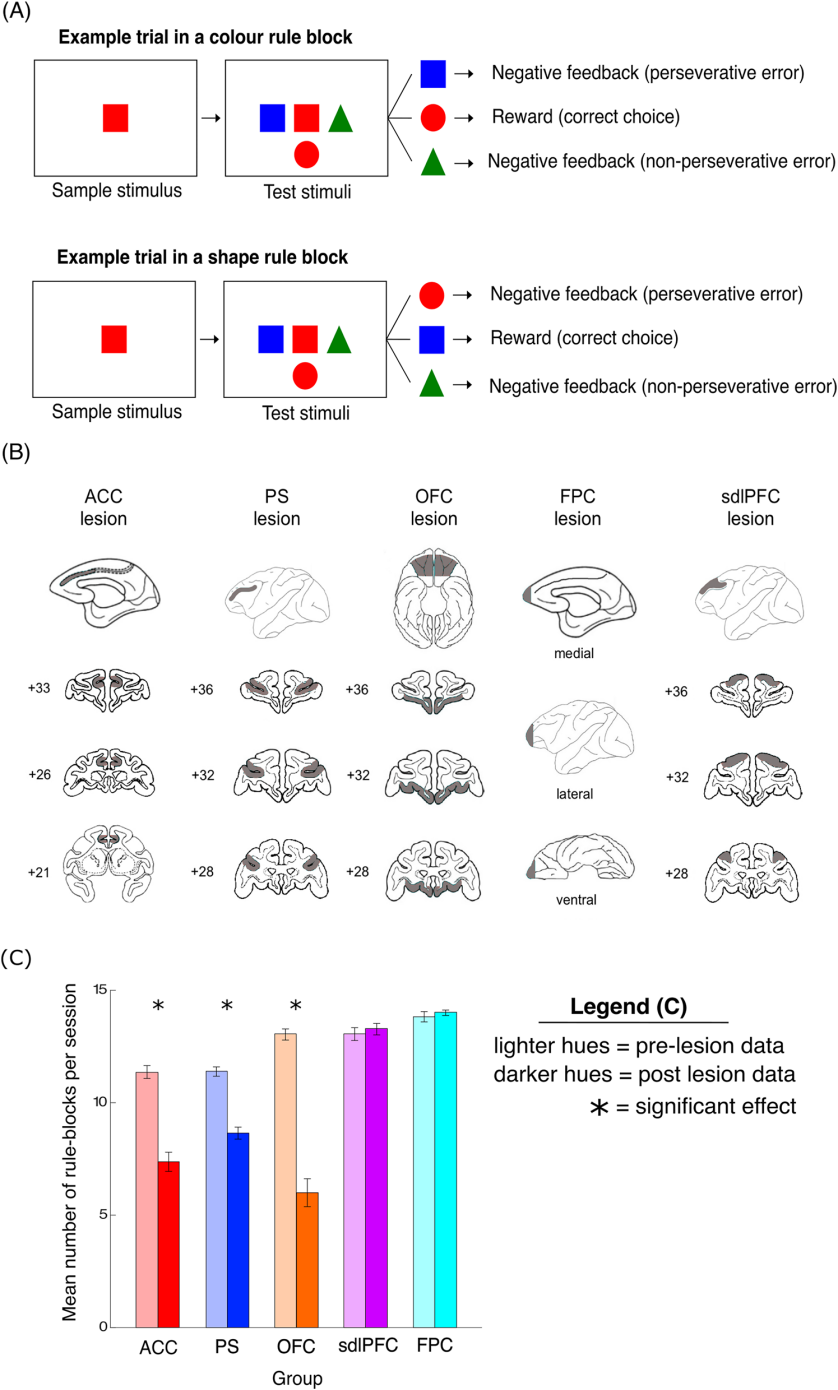
Behavioral task, intended lesions, and overall lesion effect. ***A***, The key features of the WCST are depicted in this panel. Each trial begins with the presentation of a sample stimulus in the center of a touchscreen. After the monkey touches the sample stimulus, three test stimuli appear around the sample stimulus, one matching the test sample in shape, a second matching it in color, and the third not matching the sample stimulus in either color or shape. After a correct choice (choosing stimulus according to the currently reinforced matching rule), a reward pellet is delivered. Selecting a stimulus based on the incorrect matching rule is encoded as a perseverative error (no reward is delivered). Selecting the distractor stimulus which matches the sample on neither dimension is encoded as a nonperseverative error (no reward is delivered). At no point in the task is the matching rule explicitly cued, rather, monkeys must identify the reinforced rule through trial and error. ***B***, The schematic diagrams show the extent of intended lesions in different groups of monkeys; gray regions show the extent of intended lesion (all lesions were bilateral). The details of lesion methodology and lesion extent in each group are described in the Materials and Methods section. Numerals: distance in mm from the interaural plane. Adapted from Buckley et al. (2009) and Mansouri et al. (2015). ***C***, Group mean preoperative (light hues) numbers and postoperative (dark hues) numbers of rule blocks per session for the ACC (*n* = 4), PS (*n* = 4), OFC (*n* = 3), sdlPFC (*n* = 3), and FPC (*n* = 4) lesion groups. Error bars represent SEM. The mean number of rule blocks achieved across the 10 sessions in the ACC (*n* = 4), PS (*n* = 4), OFC (*n* = 3), sdlPFC (*n* = 3), and FPC (*n* = 4) lesion groups can be seen in Extended Data Figure 1-1 (pre-lesion data) and Extended Data Figure 1-2 (post-lesion data).

Eighteen macaque monkeys were trained to perform the computerized monkey analog of the WCST (main features shown in Fig. 1). On each trial, monkeys were required to select matching stimuli based on one of two possible matching rules: matching-by-color or matching-by-shape. The currently reinforced matching rule was never explicitly cued; rather, animals needed to identify the optimal matching strategy based on choice feedback alone. To maximize reward, the animal must therefore remember the matching rule it applied on the previous trial, repeat it if it was rewarded, or change its rule if choice was not rewarded. The matching rule changed (without explicit cuing) once the animal satisfied a performance criterion, namely, achieving 85% correct choice rate averaged over 20 consecutive trials.

Daily sessions consisted of 300 trials. The first rule of the day was alternated between days. Every trial started with the presentation of a sample stimulus, randomly chosen out of all possible combinations of six distinct colors (red, green, blue, cyan, magenta, yellow) and shapes (square, circle, triangle, cross, ellipse, and hexagon). Stimuli were selected randomly without replacement, until all possible stimulus combinations in the set of 36 were used. After the monkey touched the sample stimulus, three test stimuli appeared on the screen surrounding the test stimulus (i.e., to left, below, and to right). One test stimulus matched the sample in color, the second in shape, while the last stimulus was different in both color and shape. The location of the three test stimuli on the test screen was randomized between the three possible locations, and the test stimuli were selected at random with the imposed restrictions.

The monkey then selected a test stimulus by touching it within a 5 s window; otherwise an intertrial interval began before a new trial commenced. A reward pellet was delivered after every correct choice, while the stimulus remained on the screen for 1 s, followed by a 6 s intertrial interval. After incorrect choice, no reward pellet was delivered, instead a salient visual feedback for error (large white circle) was displayed on the screen for 1 s, followed by a 12 s intertrial interval.

### Experimental schedule

Animals received preoperative training on the task until they achieved a proficiency of an average of 10 rule switches per daily session. Training and shaping progressed through a series of phases following the same training protocol in both testing laboratories. Pre-lesion data was composed of the last 10 consecutive daily sessions before surgery. Preoperatively, all monkeys also completed two control tasks. After surgery, monkeys rested for ∼14 d before once again participating in the series of control tasks and after that 10 more consecutive sessions of the WCST were analyzed in this study (control animals also rested a corresponding time between the two stages of testing).

### Preliminary training

Animals received preoperative training on the task until they achieved a proficiency of an average of 10 rule switches per daily session. Training started with the familiarization to the testing apparatus; by the end of this phase, all animals successfully learned to touch simple stimuli on the screen to obtain food reward. Second, monkeys were trained on delayed matching to sample (matching a centrally presented clip-art sample stimulus to one of two stimuli presented after a brief delay in which the sample was absent) to become acquainted with the matching principle. Next, the delay was gradually reduced until task became a simultaneous matching to sample. The number of test items was increased from two to three in the following stage, the layout and appearance of stimuli now resembling those used in the final version of the WCST analog. The subsequent stage required animals to match samples only in color or only in shape, with stimuli manipulated so that the sample and test item only matched on one dimension. Once an animal could reach criterion (85% or greater correct in single session) on this stage for both color and shape matching, they moved on to the next stage in which rules were alternated once during daily session. Next, animals were introduced to conflicting situations in which one test item matched the sample in shape, the other in color, and the third in neither dimension. Before rule changes within a daily session were introduced, animals were rewarded only for matching items based on their color until reaching a 90% criterion in a single daily session, after which they were rewarded only for matching based on shape. Only once animals could attain criterion for each new rule change in a single daily session were rule switches within a daily session introduced.

### Control tasks

All animals performed two control tasks before and after surgery to assess their basic perceptual, motor, attentional, and stimulus matching skills. In control task 1, animals completed a version of the WCST in which only one rule can be successfully applied (e.g., only one stimulus matched sample in color, no stimulus matched sample in shape). Animals therefore still had to deploy attention over many successive trials to match samples based on their color or shape, but the task did not require resolving conflict between competing matching rules. Similarly, control task 1 was a version of the WCST in which the matching rule remained constant across the entire session. The animals therefore did not have to adaptively learn changing rule values as in the main task; nevertheless, they were required to selectively maintain attention to one stimulus dimension. Performance on both control tasks was unaffected by any of the lesions investigated in this study (Buckley et al., 2009; Mansouri et al., 2015).

### Apparatus

The task was performed in an automated test apparatus, where the subjects sat unrestrained in a transport cage within reach of a touch-sensitive screen. The stimuli (5–6 cm in size) were presented on a black screen, with the center-to-center distance between test items being 15 cm. Reward pellets (190 mg) were automatically delivered into a food well on one side of the screen in response to correct choices; this was accompanied by an audible click. An infrared camera was installed to observe the subjects in the experimental cubicle, which was kept dark apart from the illumination from the touch screen. The experiment and data acquisition were controlled by a computer with a millisecond accuracy timer card.

### Animals

Data analyzed in the present study represents a subset of results obtained by two separate investigations published by Buckley et al. (2009) and Mansouri et al. (2015) (i.e., considering only the frontal lesioned animals from both studies) combined to obtain pre- and postoperative data from 18 monkeys in five frontal lesion groups in total: a PS group received bilateral lesions to the principal sulcus within the inferior dlPFC (*n* = 4, three males and one female); an ACC group received lesions to the anterior cingulate sulcus cortex within the medial frontal cortex (*n* = 4, four males); an OFC group received lesions to the orbitofrontal cortex (*n* = 3, three males); an sdlPFC group received lesions to the superior dorsolateral prefrontal cortex (*n* = 3, three males); and an FPC group received bilateral lesions to the frontal polar cortex (*n* = 4, four males). Some of the animals were *Macaca mulatta* (*n* = 7) and some *Macaca fuscata* (*n* = 11); our previously published analyses revealed no significant performance differences between the two species on this task (Buckley et al., 2009).

Data for animals trained in UK was obtained in compliance with the UK Animals (Scientific Procedures) Act 1986 and approved by Oxford's Animal Care and Ethical Review Committee. Data for animals trained in Japan was obtained in compliance with the guidelines of the Japanese Physiological Society and approved by RIKEN's Animal Experiment Committee. All animal training, surgery, and experimental procedures were the same in both laboratories.

### Surgery

The operations were performed in sterile conditions with the aid of an operating microscope and the same surgeon performed all operations for consistency of approach. Steroids (methylprednisolone, 20 mg/kg) were given (i.m.) the night before surgery, and three doses were given 4–6 h apart (i.v. or i.m.) on the day of surgery, to protect against intraoperative edema and postoperative inflammation. Each monkey was sedated on the morning of surgery with ketamine (10 mg/kg) or with ketamine (10 mg/kg), xylazine (0.5 mg/kg), and/or midazolam (0.25 mg/kg), intramuscularly. Once sedated, the monkey was given atropine (0.05 mg/kg) to reduce secretions, antibiotic (amoxicillin, 8.75 mg/kg) for prophylaxis of infection, opioid (buprenorphine 0.01 mg/kg, i.v., repeated twice at 4–6 h intervals on the day of surgery, i.v. or i.m.) and nonsteroidal anti-inflammatory (meloxicam, 0.2 mg/kg, i.v.) agents for analgesia, and an H2 receptor antagonist (ranitidine, 1 mg/kg, i.v.) to protect against gastric ulceration as a side effect of the combination of steroid and nonsteroidal anti-inflammatory treatment. The head was shaved and an intravenous cannula put in place for intraoperative delivery of fluids (warmed sterile saline drip, 5 ml/h/kg). The monkey was moved into the operating theater, intubated, placed on isoflurane anesthesia (1–2.75%, to effect, in 100% oxygen), and then mechanically ventilated. Adjustable heating blankets allowed maintenance of normal body temperature during surgery. Heart rate, oxygen saturation of hemoglobin, mean arterial blood pressure, end tidal CO_2_, body temperature, and respiration rate were monitored continuously throughout surgery. The monkey was placed in a head-holder and the head cleaned with alternating antimicrobial scrub and alcohol and draped to allow a midline incision. The skin and underlying galea were opened in layers. The temporal muscles were retracted to expose the skull surface, and a bone flap was turned to allow access to the desired lesion site with the craniotomy extended with rongeurs as necessary. The dura was cut and reflected to expose the cortex, and the lesion was made in the intended site by aspiration. When the lesion was complete, the dura was drawn back or sewn, the bone flap was replaced and held with loose sutures, and the skin and galea were closed in layers. The monkey was removed from the head-holder and anesthesia discontinued. The monkey was extubated when a swallowing reflex was observed, returned to the home cage, and monitored continuously until normal posture was regained (usually within 10 min). Nonsteroidal anti-inflammatory analgesic (meloxicam, 0.2 mg/ kg, oral) and antibiotic (8.75 mg/kg, oral) treatment continued following surgery in consultation with veterinary staff, typically for 5 d. The operated monkeys rested for ∼14 d after surgery before beginning postoperative training.

### Histology

After the conclusion of all behavioral experiments, the animals with ablations were sedated, deeply anesthetized, and then perfused through the heart with saline solution (0.9%), which was followed by formol saline solution (10% formalin in 0.9% saline solution). The brains were blocked in the coronal stereotaxic plane posterior to the lunate sulcus, removed from the skull, allowed to sink in sucrose formalin solution (30% sucrose, 10% formalin), and sectioned coronally at 50 μm on a freezing microtome. Every 10th section through the temporal lobe was stained with cresyl violet and mounted. When referring to cytoarchitecturally defined regions in the lesion descriptions below, we have adopted the nomenclature and conventions of Petrides and Pandya (Petrides and Pandya, 1994, 1999). The intended lesion extents (Fig. 1*B*), and actual lesion extents in individual animals (see previous publications for detailed individual animal lesion reconstructions, photomicrographs of some selected stained sections, and MRI scans of some sections (Mansouri et al., 2007, 2014, 2015, 2024; Buckley et al., 2009) were constructed on standard drawings based upon those provided by the Laboratory of Neuropsychology at NIMH. The lesion extents were largely as planned as assessed by microscopic inspection of aforementioned postmortem histological sections (or in some cases MRI scans) and unintended damage to underlying white matter was minor and was not bilaterally symmetrical. Details on each lesion group are provided below.

#### Anterior cingulate cortex lesion

The intended extent of the anterior cingulate cortex (ACC) lesion (Fig. 1) included the cortex within the dorsal and ventral banks of the anterior cingulate sulcus (areas 24c, 24c’), with the caudal limit of the lesion in the cingulate sulcus being an imaginary line drawn through the midpoint of the precentral dimple; the lesion extended rostrally for the full extent of the cingulate sulcus. In the ACC group, the lesions were complete and within the intended boundaries apart from in one macaque (ACCs 3 in Buckley et al., 2009), whose lesion was larger than intended in one hemisphere, and in another macaque (ACCs 4 in Buckley et al., 2009), where the lesion was not extended quite as far posteriorly as in the other three animals in order to avoid damaging ascending branches of the anterior cerebral artery observed to be present at that level of the intended lesion in that animal.

#### Principal sulcus lesion

The intended extent of the principal sulcus (PS) lesion (Fig. 1*B*) included all of the cortex in both banks, and in the fundus, of the PS along its entire anterior-posterior extent; the lesion also extended to include cortex 2–3 mm dorsal and ventral to the lips of the PS. Thus the PS lesion includes primarily the middle portion of areas 46 and 9/46. All four of the PS lesions were as intended.

#### Orbitofrontal cortex lesion

The intended extent of the orbitofrontal cortex (OFC) lesion (Fig. 1*B*) included at its lateral extent, the cortex in the medial bank of the lateral orbital sulcus; the lesion included all of the cortex between the medial and lateral orbital sulci and also extended medially until the lateral bank of the rostral sulcus. The anterior extent of the lesion was an imaginary line drawn between the anterior tips of the lateral and medial orbital sulci, and the posterior extent was an imaginary line drawn just anterior to the posterior tips of these two sulci. The intended lesion therefore included areas 11, 13, and 14 of the orbital surface and does not extend posteriorly into the agranular insula (Carmichael and Price, 1994). Microscopic examination of the stained sections confirmed that none of the OFC-lesioned animals sustained any bilateral damage outside the area of the intended region; however, two animals sustained extremely slight unilateral damage beyond the intended lateral boundary of the lesion (OFC2 and OFC3 in Buckley et al., 2009). Also, in all three animals the lesions did not extent as far medially as intended.

#### Frontopolar cortex lesion

The intended extent of the frontopolar cortex (FPC) lesion (Fig. 1*B*) was designed to include all cortex (i.e., on lateral, medial, dorsal, and ventral surfaces) anterior to an imaginary line drawn at 2 mm posterior to the rostral tip of the PS. The lesion targeted area 10. Microscopic examination of the stained sections confirmed complete lesions of the entire extent of the cortex in the frontal pole, with no damage outside of the intended region.

#### Superior dorsolateral prefrontal cortex lesion

The intended extent of the superior dorsolateral prefrontal cortex (sdlPFC) lesion (Fig. 1*B*) was designed to include the cortex on the dorsolateral aspect of the PFC extending up to midline (i.e., lateral area 9 and the dorsal portions of areas 46 and 9/46) but excluding ventrally situated dlPFC cortex that lay within the area of the PS lesion described above; the lesion excluded posteriorly located premotor areas 8A, 8Bd, and 8Bv, nor did it extend anteriorly into area 10. Microscopic examination of the stained sections confirmed all three of the sdlPFC lesions were as intended.

#### Comment on similarity in damage to white matter tracts

Three of these lesions (PS, sdlPFC, and FPC) have regions which are adjacent to each other on the lateral surface of the brain and one question that arises in interpreting the effects of lesions is whether there is likely to be, regardless of the distinct gray matter damage, similar or differential damage to white matter tracts by these lesions. Naturally, white matter fibers that course through the regions will always be compromised by aspiration lesions to those cortical areas. We also have to consider white matter tracts close to and below the cortex. Our careful and gradual aspiration lesion approach intends to minimize white matter damage below the cortex. So while we generally expect some inadvertent damage to white matter tracts that course underneath the cortex, and cannot rule it out, we expect it to be minimal, and when it does happen, it occurs sporadically and asymmetrically. Taking into account experts’ analyses of the frontal lobe association tracts (Schmahmann and Pandya, 2006; De Schotten et al., 2012) we previously published very detailed accounts of the similarities and differences in white matter tract disturbance anticipated to follow PS lesions versus FPC lesions (Ainsworth et al., 2022) and sdlPFC lesions (Kwok et al., 2019). Here we summarize the broad conclusions again. Firstly, in comparing FPC lesions with PS lesions, we note that fibers coursing to FPC (due to its location at the most rostral part of PFC) terminate in FPC and so lesions to FPC (unlike lesions to other prefrontal cortical regions) do not interrupt white matter fibers coursing through it to other brain regions. Secondly, FPC does not merely disrupt a subset of fibers that PS lesions interrupt as would be the case if all fibers innervating FPC coursed through neighboring PS. In fact we note that very few white matter fibers innervating FPC course directly through PS to get there; this is likely to be a contributing reason then (in addition to distinct gray matter damage) to why FPC and PS lesion effects are readily distinguished in this study and others (Buckley et al., 2009; Boschin et al., 2015) and indeed may be doubly dissociated across studies (Boschin and Buckley, 2015). Thirdly, FPC and PS lesions each have markedly distinct patterns of impact upon a series of different white matter fibers and connections; some are impacted more by PS lesions and others impacted more by FPC lesions, and this fact lends us to positively expect double dissociations in the effects of FPC versus PS lesions upon network functional connectivity which we showed in a previous publication (Ainsworth et al., 2022). In short, deafferentation of FPC could not account for the differential effect upon connectivity produced by the aspiration lesions to PS, or vice versa. Turning now to consider sdlPFC lesions, we note that no major fiber bundles course in the gray matter per se through the region of the intended sdlPFC lesion (Schmahmann and Pandya, 2006; De Schotten et al., 2012). However, some local fibers exist in the gray matter at certain locations in sdlPFC, and these will have been damaged; most of these connect to sdlPFC though and only a minority innervate FPC, which is anteriorly adjacent to the sdlPFC; hence the majority of connections to FPC would remain intact.

A key way to rule out disruption of white matter fibers is to make neurotoxic lesions instead of aspiration lesions, and indeed there has been important discussion in the literature distinguishing the effects of aspiration versus neurotoxic lesions to OFC. Specifically, Rudebeck et al. (2013) concluded that OFC aspiration lesions damage the underlying white matter fascicles because fiber-sparing neurotoxic OFC lesions in macaques did not impair some tasks positively impaired by the aspiration lesions. White matter damage after OFC aspiration lesions is clearly a crucial consideration. This does not mean there are not practical disadvantages of the neurotoxic lesion method exist for OFC such as repeated significant manual manipulations of cortex with brain spoons required to allow all the very many individual neurotoxic injections that may itself cause cortical trauma. Other areas have practical limitations too; in particular, neurotoxic lesions to FPC have not yet been made in macaques as far as we are aware due to practical limitations of accessing all aspects of the FPC with needles for neurotoxic injections through craniotomies that do not extend as far forward as the very anterior lesion (itself due to the spongified nature of the anteriorly located frontal air sinus in the cranium that covers part of frontal pole cortex). However, at least in the case of FPC lesions, as discussed above, there are no such white matter fascicles or bundle that passes below FPC that distributes fibers to other cortical areas. Future work may even consider alternative methods to investigate causality; for example, both electrical stimulation of FPC (Ainsworth et al., 2024) and transcranial focal ultrasound (Mahmoodi et al., 2024) have recently targeted FPC and shown behavioral effects. It is not possible with these methods to target the entire region which is a limitation. Moreover, unlike with the more traditional lesion methods used in this study, these intervention approaches have not yet shown double dissociations within prefrontal cortex.

### Data analysis and modeling

Responses on each trial were encoded as correct choices (monkey selected item based on currently reinforced matching rule), perseverative errors (applied currently nonreinforced matching rule), or nonperseverative errors (selected distractor item). To assess lesion effects on the ability to adapt to unannounced rule change, we analyzed the proportion of perseverative and nonperseverative errors in the first 10 trials following unexpected block changes. The proportion of perseverative errors was averaged across all completed rule blocks in each session performed by monkeys in each lesion group. Separately for each lesion group (ACC, PS, OFC, sdlPFC, FPC), we conducted *t* tests on the proportion of perseverative errors pre versus post lesion at each trial number and applied a familywise error correction to correct for multiple comparisons. Significant differences between pre and post lesion performance were determined using Monte Carlo testing against pseudorandomly shuffled surrogate data and cluster corrected at *p* < 0.05. Analogous analysis was performed for the proportion of nonperseverative errors.

As the total number of trials per session was fixed, we used the average number of rule blocks completed per session to characterize lesion effects on task performance. A decrease in the number of completed rule blocks indicates impaired performance, reflecting the monkeys requiring more trials to satisfy the performance criterion necessary to trigger a rule block change.

To obtain a statistical measure of the lesion effect on number of rule blocks per session, we conducted a mixed-model repeated-measure ANOVA with one between-subject factor [lesion type (5 levels, ACC, PS, OFC, sdlPFC, FPC)] and two within-subject factors [lesion presence (two levels, pre-op vs post-op) and session number (10 levels, 1–10)]. The Greenhouse–Geisser correction was applied to account for unequal variance in the sample. Post hoc *t* tests on the estimated marginal means were conducted to identify significant main effects of lesion presence in the ACC, PS, OFC, sdlPFC, and FPC groups. Bonferroni’s correction was used to correct for multiple comparisons.

### Reinforcement learning models

To ensure the selection of a model which accurately captured our data, we fit four versions of a reinforcement learning model and compared their model fit. We first fit a standard RL model with two free parameters: learning rate and choice stochasticity. This model assumes that subjects assign expected reward values to each matching rule and update their reward expectations based on choice feedback. Choice behavior is then guided by the rule with the highest expected value. Rule values are initialized to 0.5 and on each trial only the value of the chosen rule was updated. To illustrate, following the choice of a shape rule on trial *t*, the expected values of the shape rule (vS) and color rule (vC) on the following trial were updated according to the following functions:

$$vS_{\left(t+1\right)}=vS_{t}+\alpha \cdot \delta_{t},$$

$$vC_{\left(t+1\right)}=vC_{t},$$

where *α* is the learning rate parameter and $\delta_{t}\;$is the value of the reward prediction error on trial *t*, given by the following:

$$\delta_{t}=r_{t}-vS_{t},$$

That is the difference between obtained reward (*r* = 1 on rewarded trials, *r* = 0 on unrewarded trials) and expected value for rule chosen on trial *t*. On each trial, the value of the chosen rule is therefore updated by the value of the reward prediction error scaled by learning rate. The value of the unchosen rule remains unchanged.

Value estimates were transformed into choice probabilities by adopting an observational model, assuming that subjects make probabilistic choices according to the softmax distribution:

$$pS_{t}=\frac{\exp(\beta \cdot vS_{t})}{\exp\;(\beta \cdot vS_{t})+\exp(\beta \cdot vC_{t})},$$

$$pC_{t}=1-\;pS_{t},$$

where *β* represents the choice stochasticity parameter (values between 0 and 1), with larger beta values corresponding to increased levels of noise in the decision-making process. Taking the equations together, we arrive at a full likelihood function stating the probability of obtaining our data given the model and certain combination of model parameters.

Second, we fit a feedback-dependent reinforcement learning (FD-RL) model to isolate lesion effects on rule value learning in response to positive and negative feedback. To allow us to explore changes in positive and negative feedback-dependent learning, we applied two independent learning rates to positive and negative values of $\delta_{t}$*.* Value updating was done as in the standard RL model, following the same assumptions, with the exception of the learning rate $\alpha_{f}$ indexing whether the current choice was rewarded or not:

$$vS_{\left(t+1\right)}=vS_{t}+\alpha_{f}\cdot \delta_{t},$$

$$vC_{\left(t+1\right)}=vC_{t}.$$

Transformation of value estimates into categorical choices was done using the same equation as for the standard RL model. Altogether, the FD-RL model yielded three free parameters: positive learning rate (positive alpha), negative learning rate (negative alpha), and choice stochasticity (beta).

Third, we added an additional forgetting function to the standard RL model to capture effects of gradual rule value decay on choice behavior. In this forgetting RL model, value updating was done as in the standard model, as was the transformation of value estimates into categorical choices. Value decay of the shape and color rule was modeled as follows:

$$vS_{t}=vS_{t}+\varepsilon \cdot (0.5-vS_{t}),$$

$$vC_{t}=vC_{t}+\varepsilon \cdot (0.5-vC_{t}),$$

where *ε* represents the decay rate parameter (values between 0 and 1), with larger values representing a greater rate of rule value decay toward its initial value of 0.5. With the learning rate (alpha) and choice stochasticity parameter (beta), this yielded three free parameters.

Finally, we fit a hybrid Pearce–Hall (PH) model (Li et al., 2011), which includes an associability parameter modulating trial-by-trial learning rate as a function of the absolute magnitude of reward prediction error on previous trials in addition to a constant learning rate parameter. On each trial, the value of the currently chosen rule (in this example shape) is updated as follows:

$$vS_{\left(t+1\right)}=vS_{t}+\alpha_{t}\cdot \kappa \cdot \delta_{t},$$

$$vC_{\left(t+1\right)}=vC_{t},$$

where *κ* is the constant learning rate parameter optimized for each session (values between 0 and 1), $\delta_{t}$ is the reward prediction error denoting the difference between expected and actually obtained reward value, and $\alpha_{t}$ is the associability parameter. The associability parameter is initialized to 1 and updated on each trial according to the unsigned value of the reward prediction error:

$$\alpha_{\left(t+1\right)}=(1-\eta )\cdot \;\alpha_{t}+\eta \cdot |\delta_{t}|,$$

where η is a decay parameter accounting for the gradual update in associability across trials. Altogether, the Pearce–Hall RL model therefore has three free parameters: constant learning rate, associability parameter, and decay parameter.

### Analyses of model fit

For each session, each model type was fit independently to optimize free parameters. We fit the model for each session with a range of parameter combinations and maximized the likelihood of choices to arrive at optimum parameter values for each session. Akaike information criterion (AIC) was used to compare the optimized maximum likelihood obtained for each model in each session, accounting for the number of free parameters in each model. AIC values were transformed into pseudo *r*^2^ values as a measure of model fit (McFadden, 1974; Ciranka et al., 2022).

To identify which model best characterized the animals’ choice pre-lesion behavior, we conducted a mixed-model repeated-measure ANOVA on pre-lesion model fits with a between-subject factor [lesion type (five levels, ACC, PS, OFC, sdlPFC, FPC)] and two within-subject factors [model type (four levels, standard RL, FD-RL, forgetting RL, PH) and session number (10 levels, 1–10)]. The Greenhouse–Geisser correction was applied to account for unequal variance in sample. Post hoc *t* tests on the estimated marginal means were conducted to determine whether any model yielded a significantly better model fit than the standard RL model. Bonferroni’s correction was applied to *t* tests to correct for multiple comparisons.

### Optimization of model parameters

For the remainder of our analyses, we focused on the FD-RL model. For each session, the values of the beta, positive alpha, and negative alpha parameters corresponding to the best fitting model were noted. To characterize the effect of lesion on model fit, we conducted a mixed-model repeated-measure ANOVA with a between-subject factor [lesion type (five levels, ACC, PS, OFC, sdlPFC, FPC)] and two within-subject factors [lesion presence (two levels, pre-op vs post-op) and session number (10 levels, 1–10)]. This ANOVA was used to examine changes in the best fitting beta values, positive alpha values, and negative alpha values, respectively. The Greenhouse–Geisser correction was applied to account for unequal variance in sample. Post hoc *t* tests on the estimated marginal means were conducted to identify significant main effects of lesion type in the ACC, PS, OFC, sdlPFC, and FPC groups. Bonferroni’s correction was applied to *t* tests to correct for multiple comparisons.

### Analyses of choice behavior relative to trial-by-trial value of the reward prediction error

For all sessions, we obtained trial-by-trial values of the reward prediction error predicted by the best fitting model for a given session. Trials with large positive (0.75 to 1), large negative (−1 to −0.75), small positive (0 to 0.25), and small negative (−0.25 to 0) RPEs were selected. For each RPE value, we then determined the probability of repeating a color/shape choice on the trials which immediately followed the critical large positive/large negative/small positive/small negative RPE trials.

To determine the lesion effect on probability of repeating choices after a predefined RPE value, we conducted a mixed-model repeated-measure ANOVA with one between-subject factor [lesion type (five levels, ACC, PS, OFC, sdlPFC, FPC)] and two within-subject factors [lesion presence (two levels, pre-op vs post-op) and session number (10 levels, 1–10)]. This ANOVA was calculated for the probability of repeating a choice after small positive, small negative, high positive, and high negative RPEs separately. The Greenhouse–Geisser correction was applied to account for unequal variance in sample group. Post hoc *t* tests on the estimated marginal means were conducted to identify significant main effects of lesion type in the ACC, PS, OFC, sdlPFC, and FPC groups. Bonferroni’s correction was used to correct *p* values for multiple comparisons.

## Results

### Effects of distinct frontal lesions on WCST performance

We obtained behavioral data (previously published in other forms: Buckley et al., 2009; Mansouri et al., 2015) from 18 monkeys before and after frontal lesions performing the macaque analog of the WCST. Animals were trained to select matching sample stimuli based on their color or shape on a touchscreen with a reward for each correct response. At no point in the task was the currently reinforced rule explicitly cued. Instead, monkeys had to learn which matching rule was currently reinforced based on choice feedback, and then they had to continue applying the identified matching rule across trials until the rule changed unexpectedly once a predefined performance criterion (85% over 20 consecutive trials) was reached.

After task training (see Materials and Methods), monkeys were split in stages (see original studies) into lesion or unoperated control groups of matched abilities and accordingly received aspiration lesions (Fig. 1*B*) to either the ACC (*n* = 4), PS (*n* = 4), OFC (*n* = 3), sdlPFC (*n* = 3), or FPC (*n* = 4). None of the lesion groups were impaired on control tasks involving color and shape matching without rule switches, suggesting that their perceptual, motor, and attentional abilities were intact. In this study, to be consistent across animals, the pre-lesion data were composed of the last 10 consecutive daily sessions before surgery. After recovering from surgery, all monkeys performed 10–15 more sessions of the WCST, and we analyzed the first 10 consecutive sessions of these to obtain post-lesion data for this study.

10.1523/ENEURO.0117-25.2025.f1-1 Figure 1-1 Bar chart showing the pre-operative number of rule blocks achieved per session, averaged across all monkeys in the ACC, PS, OFC, sdlPFC and FPC lesion groups. Error bars represent SEM. Download Figure 1-1, TIF file.

**Figure 2.**
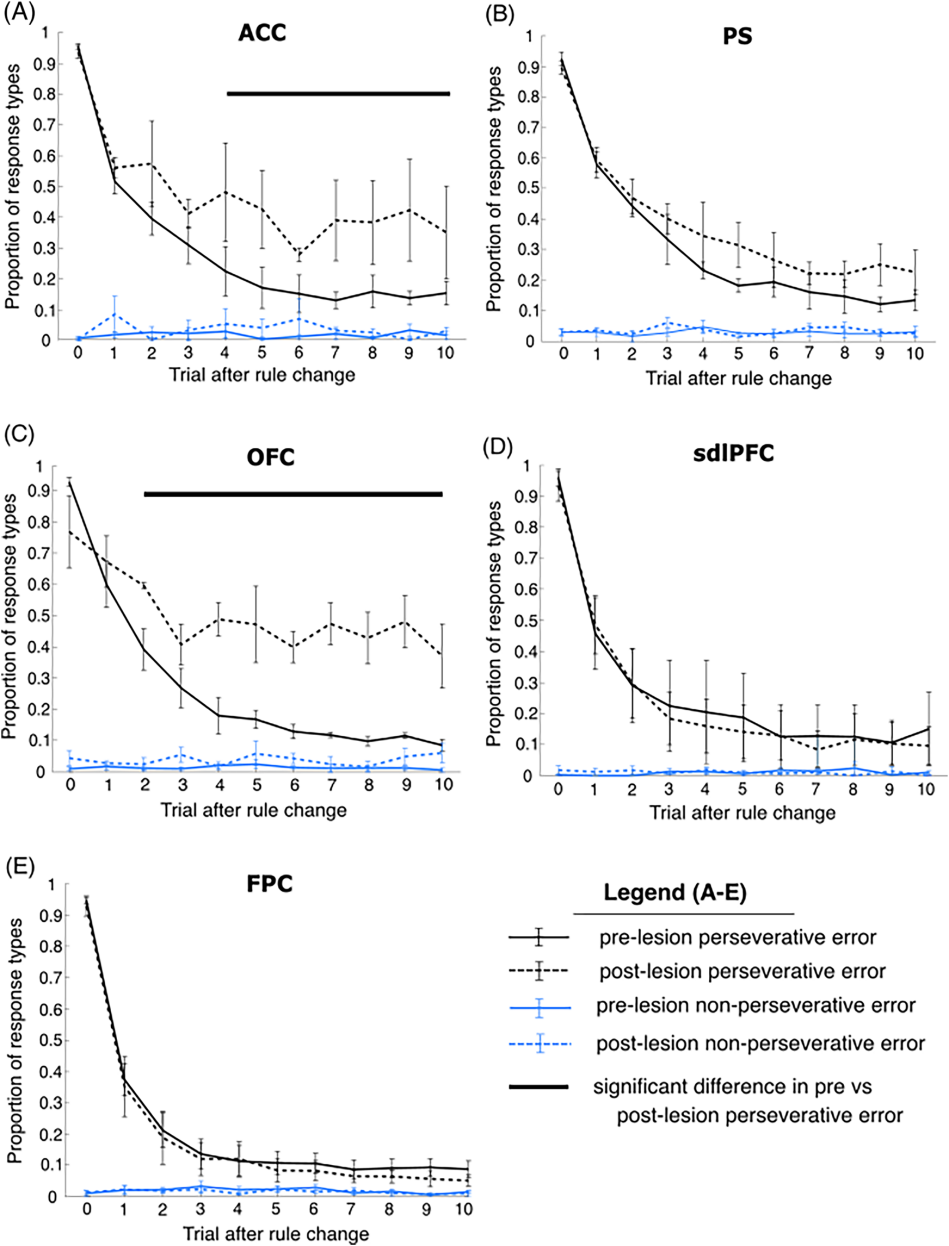
Error responses following rule change. For each of the five groups, (***A***) ACC lesion, (***B***) PS lesion, (***C***) OFC lesion, (***D***) sdlPFC lesion, and (***E***) FPC lesion, the proportion of pre- and postoperative perseverative errors (black) and the proportion of pre and postoperative nonperseverative errors (blue) on the first 10 trials following the first trial with an unexpected rule change in the WCST. Error bars represent SEM. Black bar represents trials in which there was a significant difference (*p* < 0.05) between preoperative and postoperative proportion of perseverative errors (all comparisons cluster corrected at *p* < 0.05 for multiple comparisons).

10.1523/ENEURO.0117-25.2025.f1-2 Figure 1-2 Bar chart showing the post-operative number of rule blocks achieved per session, averaged across all monkeys in the ACC, PS, OFC, sdlPFC and FPC lesion groups. Error bars represent SEM. Download Figure 1-2, TIF file.

As previously reported, after the lesions were introduced, the ACC, OFC, and PS groups were significantly impaired on the WCST analog, but the sdlPFC and FPC groups were not (Buckley et al., 2009; Mansouri et al., 2015). To further assess and compare lesion effects on WCST performance here across these five frontal groups, we analyzed the mean number of rule blocks achieved per session (Fig. 1*C*). Because the number of trials in each testing session was fixed, the number of rule block switches was taken as a suitable measure of overall performance in the WCST. A repeated-measure ANOVA with one between-subject factor [lesion type (five levels corresponding to the five lesions: ACC, PS, OFC, sdlPFC, FPC] and two within-subject factors [lesion presence (two levels: pre-op vs post-op) and session (10 levels corresponding to each test session)] revealed a main effect (*F*_(9,117)_ = 171.7, *p* = 1.77 × 10^−11^) of lesion presence on the number of rule blocks achieved per session (full ANOVA output can be seen in Table 1). Post hoc tests on the estimated marginal means revealed a significant effect of lesion presence in the ACC (11.35 ± 0.29 vs 7.38 ± 0.43 block changes pre and post lesion, *p* = 0.0062, Bonferroni’s correction for multiple comparisons), PS (11.40 ± 0.21 vs 8.65 ± 0.27 block changes pre and post lesion, *p* = 0.042), and OFC (13.07 ± 0.25 vs 6.00 ± 0.62 block changes pre and post lesion, *p* = 0.00024) groups. In contrast, lesions to the sdlPFC (13.01 ± 0.28 vs 13.3 ± 0.26 block changes pre and post lesion, *p* = 0.87) and FPC (13.82 ± 0.23 vs 14.03 ± 0.12 block changes pre and post lesion, *p* = 0.87) had no significant effect on the number of rule blocks per session.

**Table 1.** Full output of ANOVA on number of blocks completed per session with lesion presence (two levels, pre vs post lesion) and session (10 levels, 1–10) as the within-subject variables and lesion type (five levels, ACC, PS, OFC, sdlPFC, and FPC) as the between-subject variable

	Sum of squares	df	Mean square	*F*	*p* (GG corrected)
Lesion type	1,377.300	4	344.300	1.600	0.240
Lesion presence	44,966.000	9	4,996.200	171.700	1.77 × 10^−11^
Session	89.100	9	9.900	4.000	0.005
Lesion type × lesion presence	2,054.100	36	57.060	2.000	0.130
Lesion type × session	84.600	36	2.400	0.900	0.530
Lesion presence × session	45,083.000	20	2,254.100	152.700	6.00 × 10^−13^
Lesion presence × lesion type × session	2,182.200	80	27.300	1.800	0.130

As the results of the ANOVA revealed a significant interaction between lesion presence and session number on the number of rule blocks achieved per session (*F*_(20,260)_ = 152.7, *p* = 6.002 × 10^−13^), we further investigated the possibility of a post-lesion learning effect across sessions. For each post lesion group separately, we conducted linear regressions using the session number at the predictor variable and the number of blocks achieved per session as the dependent variable. A significant regression was not found in the ACC (*F*_(1,38)_ = 2.3, *p* = 0.14), PS (*F*_(1,38)_ = 0.99, *p* = 0.33), OFC (*F*_(1,38)_ = 4.0, *p* = 0.056), sdlPFC (*F*_(1,38)_ = 0.28, *p* = 0.60), and FPC (*F*_(1,38)_ = 0.23, *p* = 0.63) lesion groups. We therefore cannot draw any conclusions about lesion differences in speed of postoperative task performance recovery based on our results.

To determine whether each frontal lesion caused differing impairments in the animal's adaptation to WCST rule shifts, we analyzed choice behavior across the 10 trials following rule block changes. Responses on each trial were encoded as correct choices (monkey selected item based on currently reinforced matching rule), perseverative errors (applied currently nonreinforced matching rule), or nonperseverative errors (selected distractor item), for mean proportion of error types across trials immediately following block change Figure 2. Across all lesion groups, the majority of errors were perseverative (91.75% pre-op, 91.03% post-op); animals therefore rarely selected the stimulus which matched sample items on neither dimension.

To assess lesion effects on the ability to adapt to unannounced rule change, we analyzed the response types across the first 10 trials following the trial of the rule block changes. While OFC lesions significantly increased the proportion of perseverative errors from the second trial immediately following an unexpected rule change, ACC lesions only had an effect on perseverative error rate starting at the fourth trial following a block change. We found a significant difference between the proportion of perseverative errors pre versus post lesion in the second to the 10th trial after block change in the OFC group [*t* test *p* < 0.001, cluster corrected at *p* = 0.05, peak effect (*p* = 5.13 × 10^−4^) at the ninth trial following block change, with mean probability of perseverative error 0.12 ± 0.0090 vs 0.48 ± 0.084 for pre and post lesion]. In the ACC lesion group, we found a significant effect of lesion presence on the proportion of perseverative errors in the fourth to 10th trials relative to the nearest block change (*t* test *p* < 0.001, cluster corrected at *p* = 0.05, peak effect at trial 7 with mean probability of preservative error 0.22 ± 0.087 vs 0.48 ± 0.13 for pre and post lesion). In contrast, PS, sdlPFc, and FPC lesions did not significantly affect choice performance across any of the first 10 trials following block change (*p* > 0.05 for all trials in sdlPFC, FPC, and PS lesion groups). None of the lesions significantly affected the proportion of nonperseverative errors in the first 10 trials following block change (*p* > 0.05 for all trials in ACC, PS, OFC, sdlPFC, and FPC lesion groups).

The high proportion of perseverative errors in the very first trial following block change across all pre and post lesion groups confirmed that animals could not reliably predict the upcoming change in reward contingency and instead continued applying the previously reinforced matching rule. The gradual decrease in perseverative errors following block change suggests that a single unexpected reward is generally insufficient to inform animals of rule change. Rather, choice feedback is integrated with recent reinforcement history to shape response behavior.

### Model selection

We compared the model fit of four different versions of RL models to ensure our selected model optimally captured moneys’ rule value learning and choice behavior in the WCST. First, a standard two-parameter RL model which updated the value of the chosen rule on each trial by scaling RPE by a learning rate parameter. Second, a feedback-dependent RL model (FD-RL) which included a feedback-dependent learning rate parameter to account for possible distinctions in rule value learning following positive and negative feedback. Third, a forgetting RL model in which a value discounting function was added to the standard RL model to account for possible rule value decay across trials. Lastly, we fit a hybrid Pearce–Hall (PH) model (Li et al., 2011) with a variable learning rate scaled by the absolute magnitude of past RPEs capturing attention modulation during learning.

**Figure 3.**
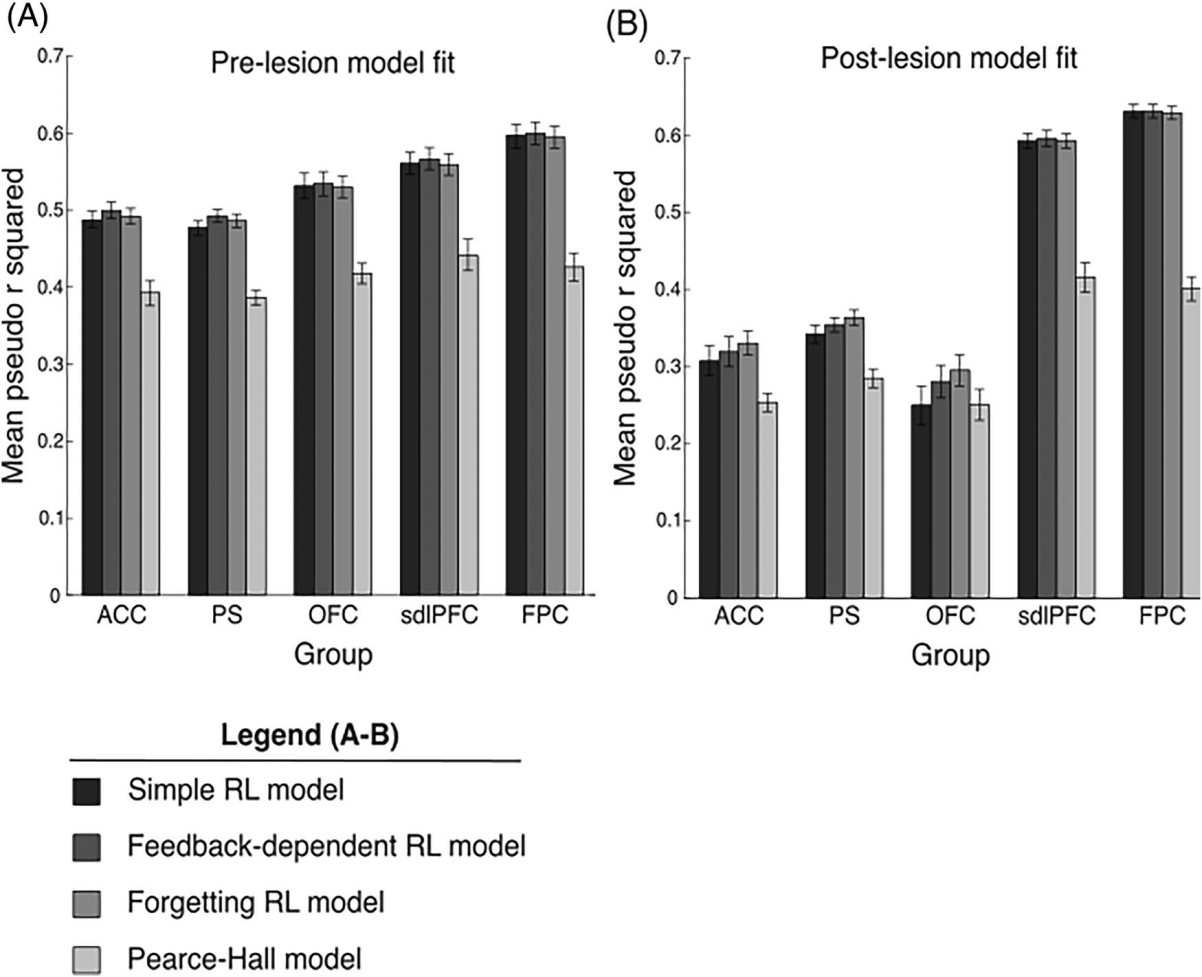
Comparison of model fit. Bar charts showing the mean pseudo *r* squared values, representing model fit, of the simple RL model, the feedback-dependent RL model, the forgetting RL model, and the Pearce–Hall model across the five groups (ACC, PS, OFC, sdlPFC, FPC) before (***A***) and after lesion (***B***). Error bars represent SEM.

Our results show that the FD-RL model was most suitable for examining the lesion effects on WCST performance, as it was the only tested model which fit the pre-lesion data significantly better than the standard RL model (Fig. 3*A*). A mixed-model repeated-measures ANOVA on pre-lesion model fit with lesion type as the between-subject factor (ACC, PS, OFC, sdlPFC, FPC) and model type (simple RL, FD-RL, forgetting RL, PH) and session number (1–10) as the within-subject factor revealed a significant main effect of model type on model fit (*F*_(9,117)_ = 177.3, *p* = 2.05 × 10^−9^). Full ANOVA output can be seen in Table 2. Post hoc testing on the estimated marginal means revealed that pre-lesion model fit was significantly better for the FD-RL model than the standard RL model (pseudo *r*^2^ value = 0.54 ± 0.039 and 0.55 ± 0.038 for the standard RL model and FD-RL model, respectively, *p* = 0.0035). The model fit of the PH model was significantly worse than the standard RL model (pseudo *r*^2^ value = 0.54 ± 0.039 and 0.41 ± 0.024 for the standard RL model and PH model, respectively, *p* = 3.15 × 10^−5^). There was no significant difference between the fit of the standard RL model and the forgetting RL model (pseudo *r*^2^ value = 0.54 ± 0.039 and 0.54 ± 0.037 for the standard RL model and forgetting RL model, respectively, *p* = 0.74). Furthermore, the FD-RL model also provided the best model fit for post-lesion data out of the four model variants, as can be seen in Figure 3*B*. As our results show that the FD-RL model fits our data well relative to other models, and the inclusion of a feedback-dependent learning rate parameter enables the isolation of distinct lesion effects on the ability to learn rule values in the WCST, we henceforth analyzed the lesion effects on the fit and model parameter values of the FD-RL model.

**Table 2.** Full output of ANOVA on model fit (pseudo *r*^2^ values) with model (four levels: standard RL model, FD-RL model, forgetting RL model, PH model) and session number (10 levels, 1–10) as the within-subject variables and lesion type (five levels, ACC, PS, OFC, sdlPFC, and FPC) as the between-subject variable

	Sum of squares	df	Mean square	*F*	*p* (GG corrected)
Model	185.940	9	20.660	177.310	2.05 × 10^−09^
Lesion type	1.323	4	0.331	0.336	0.849
Session	0.105	9	0.012	0.552	0.746
Model × lesion type	1.631	36	0.045	0.389	0.824
Model × session	186.060	40	4.651	148.720	1.40 × 10^−11^
Lesion type × session	0.671	36	0.019	0.881	0.615
Model × lesion Type × session	2.403	160	0.015	0.480	0.812

### Effect of distinct prefrontal lesions on FD-RL model parameters

**Figure 4.**
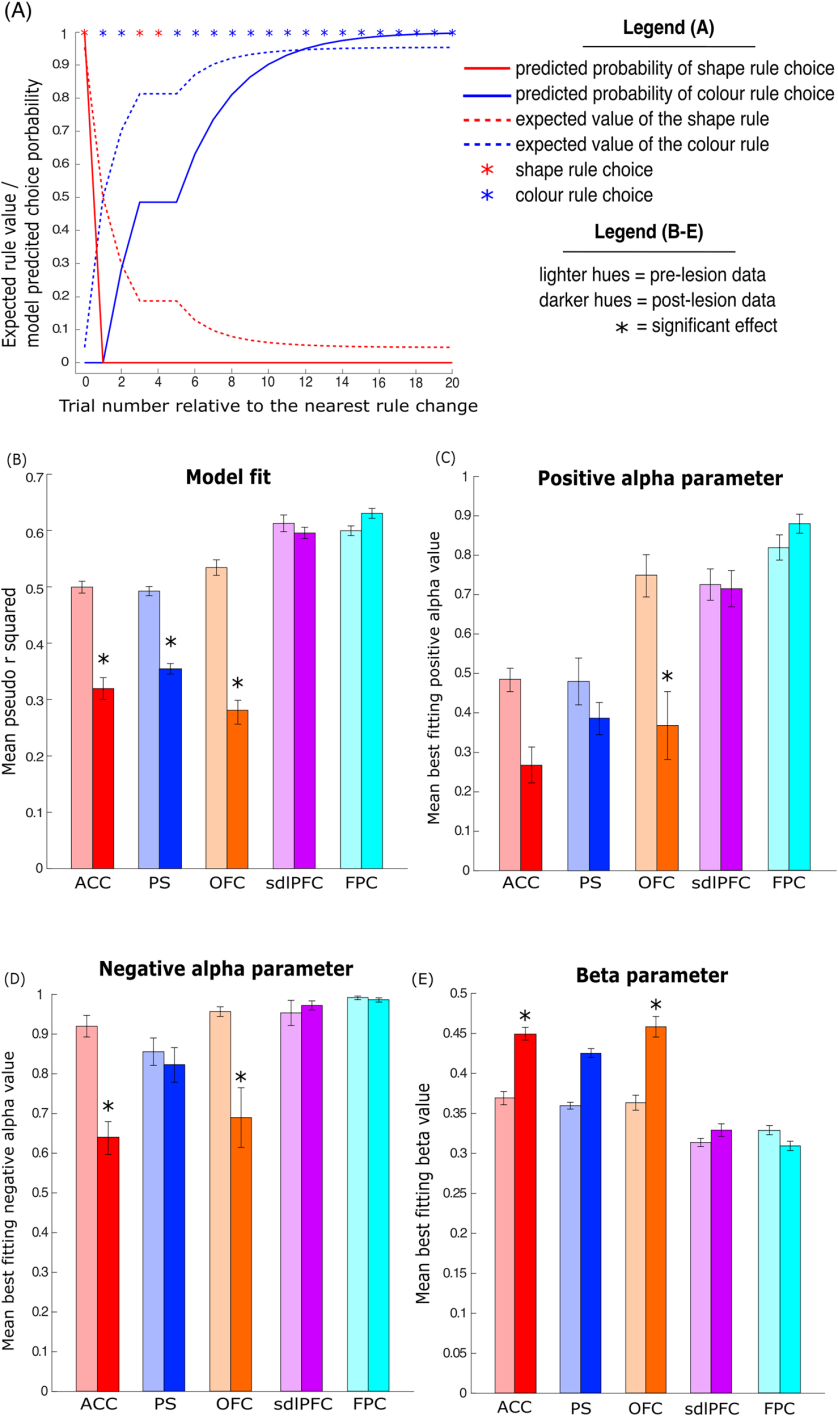
Effects of frontal lesions on model fit and best fitting model parameters. ***A***, Example data showing trial by trial model-predicted expected rule value (solid line) and model-predicted choice probability (broken line) in one color rule block of the WCST. After an unexpected rule change (trial 0), the expected value of the currently rewarded color rule increases, while the value of the unrewarded shape rule decreases. On each trial, the change in expected rule value corresponds to the current value of the reward prediction error (difference between expected and actually obtained reward) scaled by the session-wise best fitting value of the learning rate parameter. The relationship between current rule value and probability of making a choice in favor of the given rule is informed by the best fitting value of model parameter beta, reflecting choice stochasticity. ***B–E***, Bar charts showing the group mean model fit, as well as the mean parameters of the best fitting models, obtained from RL models fit independently to all behavioral sessions for pre (light hues) and post (dark hues) lesion groups. For all data shown errors bars denote SEM. ***B***, Overall model fit quantified using pseudo *r*^2^, (***C***) positive alpha parameter, (***D***) negative alpha parameter, and (***E***) choice stochasticity parameter beta.

Our data suggest that while overall WCST performance was impaired following ACC, PS, and OFC, the effect on rule learning may differ in each case. To identify how distinct prefrontal regions affect the ability to learn rule values from positive and negative feedback and successfully apply optimal matching rules to maximize reward in the WCST, we utilized the feedback-dependent reinforcement learning model (FD-RL model). This model had three parameters: learning rate from positive feedback (positive alpha), learning rate from negative feedback (negative alpha), and choice stochasticity (beta). Specifically, our FD-RL model assumes that monkeys make choices in the WCST by assigning values to each of the matching rules, iteratively updating predicted rule values based on choice feedback, and select stimuli by applying the highest valued matching rule (Fig. 4*A*). All free model parameters were optimized independently for each session before and after lesions (see Materials and Methods), yielding an average preoperative model fit of 0.545 pseudo *r*^2^.

Lesions to the ACC, PS, and OFC, which were found to significantly impair task performance, were also associated with a significantly decreased model fit (Fig. 4*B*). A repeated-measures ANOVA (with same factors and levels as described above) detected a significant main effect (*F*_(9,117)_ = 151.2, *p* = 4.57 × 10^−11^) of lesion presence (pre vs post-op) on model fit (see Table 3 for full ANOVA output). Consistent with the previous behavioral analysis post hoc testing on the estimated marginal means revealed a main effect of lesion presence on model fit in the ACC (mean *r*^2^ value of 0.50 ± 0.011 vs 0.32 ± 0.019 pre and post lesion, *p* = 0.0054, Bonferroni’s correction for multiple comparisons), OFC (mean pseudo *r*^2^ value of 0.53 ± 0.014 vs 0.28 ± 0.021 pre and post lesion, *p* = 0.0013), and PS (mean pseudo *r*^2^ value of 0.49 ± 0.0082 vs 0.35 ± 0.0093 pre and post lesion, *p* = 0.024) groups. In contrast, FPC lesions (mean pseudo *r*^2^ value of 0.60 ± 0.0086 vs 0.63 ± 0.0087 pre and post lesion, *p* = 0.58) and sdlPFC lesions (mean pseudo *r*^2^ value of 0.61 ± 0.015 vs 0.60 ± 0.010 pre and post lesion, *p* = 0.79) had no effect on model fit. Despite the decrease in model fit in the ACC, OFC, and PS groups, suggesting that these lesions may introduce additional task-related factors not captured by our model, all post-op pseudo *r*^2^ values correspond to good model fit (McFadden, 1974), signifying the suitability of our model for the task.

**Table 3.** Full output of ANOVA on model fit (pseudo *r*^2^ values) with lesion presence (two levels, pre vs post lesion) and session (10 levels, 1–10) as the within-subject variables and lesion type (five levels, ACC, PS, OFC, sdlPFC, and FPC) as the between-subject variable

	Sum of squares	df	Mean square	*F*	*p* (GG corrected)
Lesion type	3.317	4	0.829	1.741	0.201
Lesion presence	86.578	9	9.620	151.240	4.57 × 10^−11^
Session	0.130	9	0.014	2.254	0.067
Lesion type × lesion presence	4.393	36	0.122	1.918	0.136
Lesion type × session	0.184	36	0.005	0.794	0.699
Lesion presence × session	86.718	20	4.336	130.820	5.22 × 10^−13^
Lesion presence × lesion type × session	4.645	80	0.058	1.752	0.145

We next assessed lesion effects on specific model parameters to relate model components to the intact functioning of distinct brain areas. First, we analyzed how monkeys updated the expected value of the color and shape rule in response to positive and negative feedback by interrogating the preoperative and postoperative best fitting values of the learning rate parameter. The feedback-dependent RL model includes two separate learning rate parameters, positive and negative alpha, to account for possible distinctions in value updating following positive and negative feedback, respectively. While positive alpha is used to scale value updating on trials following reward delivery, negative alpha scales value updating on trials following omission of reward.

OFC lesions significantly impaired the animals’ ability to learn from positive reinforcement, as indicated by significantly lower best fitting positive alpha values in the post OFC lesion group (Fig. 4*C*). The results of a repeated-measure ANOVA (see Materials and Methods) showed a significant interaction (*F*_(36,117)_ = 3.93, *p* = 0.00027) between lesion type (ACC, PS, OFC, sdlPFC, and FPC) and lesion presence (pre-op vs post-op) on best fitting positive alpha values (see Table 4 for full ANOVA output). Post hoc testing on the estimated marginal means revealed a significant lesion effect on best fitting positive alpha values in the OFC (mean positive alpha 0.75 ± 0.054 vs 0.37 ± 0.086 for pre and post lesion, *p* = 0.0083, Bonferroni’s correction for multiple comparisons) lesion group; this effect was not significant in the ACC (mean positive alpha 0.49 ± 0.030 vs 0.27 ± 0.046 for pre and post lesion, *p* = 0.062), PS (mean positive alpha 0.48 ± 0.059 vs 0.39 ± 0.040 for pre and post lesion, *p* = 0.40), sdlPFC (mean positive alpha 0.73 ± 0.40 vs 0.71 ± 0.046 for pre and post lesion, *p* = 0.93), or FPC (mean positive alpha 0.82 ± 0.032 vs 0.88 ± 0.024 for pre and post lesion, *p* = 0.58) groups.

**Table 4.** Full output of ANOVA on model-predicted positive alpha values with lesion presence (two levels, pre vs post lesion) and session (10 levels, 1–10) as the within-subject variables and lesion type (five levels, ACC, PS, OFC, sdlPFC, and FPC) as the between-subject variable

	Sum of squares	df	Mean square	*F*	*p* (GG corrected)
Lesion type	12.028	4	3.007	7.470	0.002
Lesion presence	123.680	9	13.742	119.710	4.63 × 10^−22^
Session	0.992	9	0.110	1.606	0.178
Lesion type × lesion presence	16.233	36	0.451	3.928	0.000
Lesion type × session	2.139	36	0.059	0.866	0.619
Lesion presence × session	124.790	20	6.240	69.657	1.25 × 10^−29^
Lesion presence × lesion type × session	19.766	80	0.247	2.758	0.000

Value updating in response to negative feedback was impaired in both the ACC and OFC lesion groups, as evidenced by significantly lower best fitting negative alpha values (Fig. 4*D*). A three-way ANOVA (as above) detected a significant main effect (*F*_(9,117)_ = 297.4, *p* = 2.33 × 10^−14^) of lesion presence (pre-op vs post-op) on negative alpha values (see Table 5 for full ANOVA output). Post hoc testing on the marginal means revealed a significant lesion presence effect in the ACC (mean negative alpha 0.92 ± 0.027 vs 0.64 ± 0.042 for pre and post lesion, *p* = 0.0043, Bonferroni’s correction for multiple comparisons) and OFC (mean negative alpha 0.96 ± 0.01 vs 0.69 ± 0.075 for pre and post lesion, *p* = 0.013) groups. No significant lesion effect the on best fitting negative alpha values was detected in the PS (mean negative alpha 0.86 ± 0.035 vs 0.82 ± 0.044 for pre and post lesion, *p* = 0.69), sdlPFC (mean negative alpha 0.95 ± 0.032 vs 0.97 ± 0.012 for pre and post lesion, *p* = 0.84), and FPC (mean negative alpha 0.99 ± 0.0045 vs 0.99 ± 0.0054 for pre and post lesion, *p* = 0.94) groups.

**Table 5.** Full output of ANOVA on model-predicted negative alpha values with lesion presence (two levels, pre vs post lesion) and session (10 levels, 1–10) as the within-subject variables and lesion type (five levels, ACC, PS, OFC, sdlPFC, and FPC) as the between-subject variable

	Sum of squares	df	Mean square	*F*	*p* (GG corrected)
Lesion type	2.483	4	0.621	1.075	0.408
Lesion presence	273.660	9	30.407	297.410	2.33 × 10^−14^
Session	0.296	9	0.033	1.079	0.372
Lesion type × lesion presence	4.497	36	0.125	1.222	0.321
Lesion type × session	1.449	36	0.040	1.321	0.236
Lesion presence × session	273.940	20	13.697	199.320	1.74 × 10^−20^
Lesion presence × lesion type × session	7.418	80	0.093	1.349	0.245

Next, we analyzed the pre-op and post-op best fitting values of the inverse temperature parameter beta to identify lesion effects on choice consistency. The value of the beta parameter determines the level of noise included in the transformation of value estimates into choice probability. Higher beta values are therefore indicative of monkeys choosing the higher-value option less consistently. Analysis of the beta values obtained from the best fitting models suggest that lesions to the OFC and ACC caused animals’ choices to become less predictable and increasingly random (Fig. 4*E*). The results of a repeated-measure ANOVA revealed a main effect (*F*_(9,117)_ = 292.1, *p* = 1.024 × 10^−14^) of lesion presence (pre-op vs post-op) on best fitting beta values (see Table 6 for full ANOVA output). Post hoc tests show a significant effect of lesion presence in the ACC (mean beta value 0.37 ± 0.0082 vs 0.45 ± 0.0079 pre and post ACC lesion, *p* = 0.022, Bonferroni’s correction for multiple comparisons) and OFC (mean beta value 0.36 ± 0.0094 vs 0.46 ± 0.013 for pre and post lesion, *p* = 0.019) groups. No significant lesion presence effect on beta values was detected in the PS (mean beta value 0.36 ± 0.0042 vs 0.43 ± 0.0055 for pre and post lesion, *p* = 0.052), sdlPFC (mean beta value 0.31 ± 0.0051 vs 0.33 ± 0.0079 for pre and post lesion, *p* = 0.67), and FPC (mean beta value 0.33 ± 0.0057 vs 0.31 ± 0.0059 pre and post lesion, *p* = 0.54) groups.

**Table 6.** Full output of ANOVA on model-predicted beta values with lesion presence (two levels, pre vs post lesion) and session (10 levels, 1–10) as the within-subject variables and lesion type (five levels, ACC, PS, OFC, sdlPFC, and FPC) as the between-subject variable

	Sum of squares	df	Mean square	*F*	*p* (GG corrected)
Lesion type	0.613	4	0.153	1.176	0.366
Lesion presence	48.577	9	5.397	292.120	1.02 × 10^−14^
Session	0.024	9	0.003	0.973	0.438
Lesion type × lesion presence	0.829	36	0.023	1.246	0.325
Lesion type × session	0.068	36	0.002	0.702	0.800
Lesion presence × session	48.601	20	2.430	241.450	7.02 × 10^−19^
Lesion presence × lesion type × session	0.928	80	0.012	1.153	0.361

### Lesion effects on choice behavior relative to trial-by-trial value of the reward prediction error

**Figure 5.**
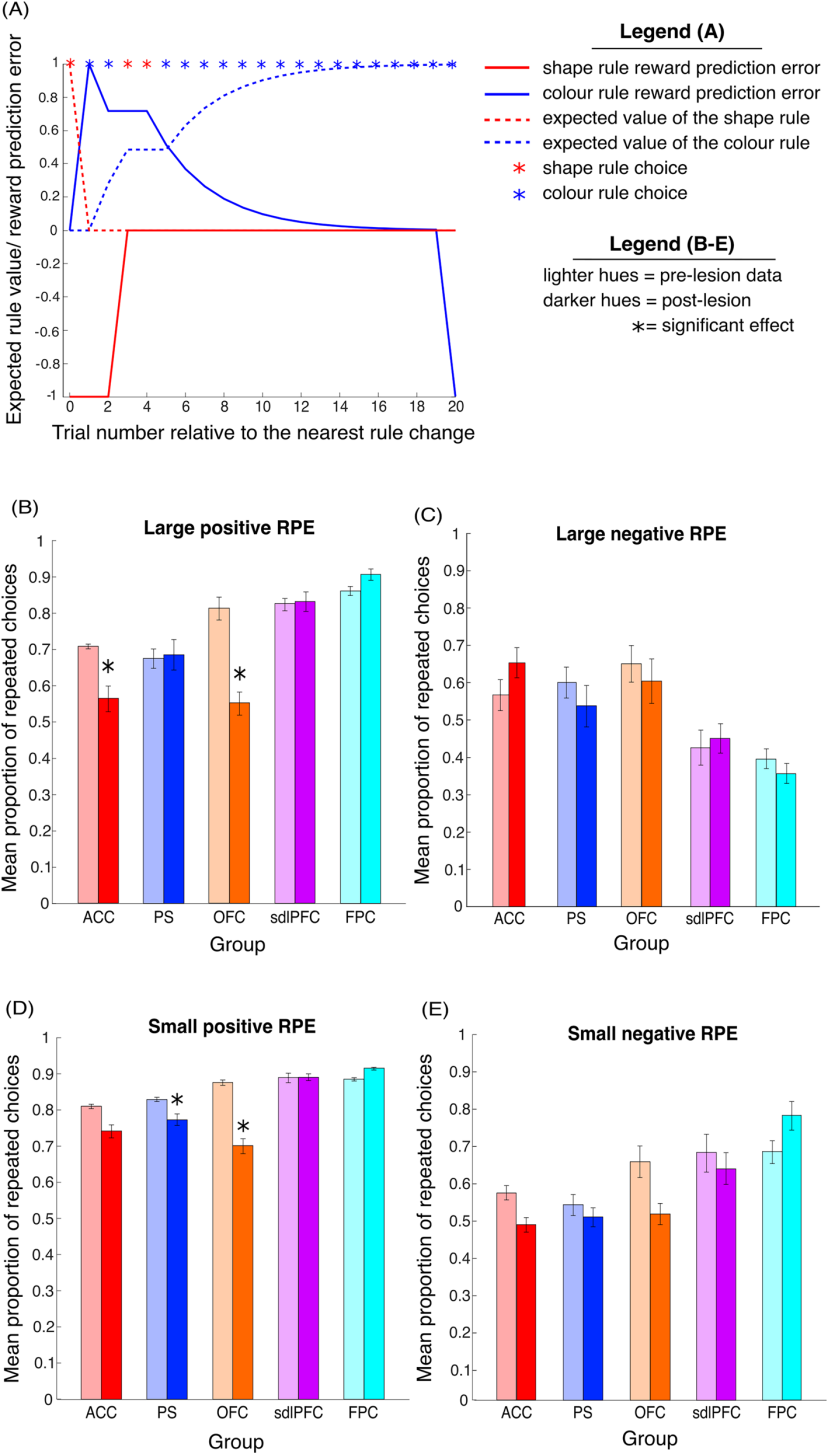
Analysis of choice behavior relative to trial-by-trial values of the reward prediction error. ***A***, Example data showing trial by trial expected rule value (broken line) and reward prediction error (solid line) in one color rule block of the WCST. After an unexpected change from shape to color rule, shape choice on trial 1 generates high negative prediction error. This drives the decrease in the expected value of the shape rule on the following trial. First correct application of the reinforced color rule (trial 2) generates a high positive prediction error, as reward delivery is surprising given the low expected value of the color rule. Consequently, the expected value of the color increases. As the value of color is learned (from trial 2 onward), color choices generate gradually smaller prediction errors. ***B–E***, Bar charts showing the mean probability of animals repeating choices split by the relative prediction error (RPE) from the previous trial across all behavioral sessions for pre (light hues) and post (dark hues) lesion groups. For all data shown errors bars denote SEM. RPE shown separately for (***B***) Large positive RPE values (0.75 to 1), (***C***) large negative RPE values (−1 to −0.75), (***D***) small positive RPE values (0 to 0.25), and (***E***) small negative RPE values (−0.25 to 0).

To further characterize the effect of distinct frontal lesions on the ability to learn rule values from choice feedback, we analyzed the animals’ choices relative to reward prediction error (RPE) obtained from the RL models. We obtained trial-by-trial model estimates of the RPE, that is, the difference between the expected value of the currently chosen rule (0–1) and the actually received reward value (1 when reward delivered, 0 when no reward delivered). Scaled by the learning rate parameter, the RPE determines the extent to which the value of a given rule should be updated on each trial to optimize choice behavior toward maximizing reward. The trial-by-trial values of the RPE and expected rule value are illustrated in Figure 5*A*.

First, we analyzed choice behavior following high RPE (RPE = 0.75 to 1) to identify how frontal lesions affect the animal's ability to adapt to surprising changes in reward value. High positive prediction errors corresponded to trials in which reward delivery was unexpected because the value of the correct rule had yet to be learned (e.g., following the first correct application of a newly reinforced rule). In contrast, high negative prediction errors arise when no reward is delivered following the application of a highly valued rule (e.g., following unexpected change in reward association after rule shift).

OFC and ACC lesion groups were significantly impaired when applying the correct rule following trials with a large positive RPE (Fig. 5*B*). We conducted a repeated-measure ANOVA on the probability of repeating a correct choice, with one between-subject factor [lesion type (ACC, PS, OFC, sdlPFC, and FPC)] and two within-subject factors [lesion presence (pre-op vs post-op) and session number (1–10)]. There was a significant interaction between lesion presence and lesion type (*F*_(36,108)_ = 3.192, *p* = 0.014; see Table 7 for full ANOVA output). The results of planned post hoc testing on the estimated marginal means corrected comparisons revealed a significant lesion effect on the probability of repeating choices following high RPE trials in the OFC (mean probability 0.81 ± 0.03 vs 0.55 ± 0.032 pre and post lesion, *p* = 0.00077, Bonferroni’s correction for multiple comparisons) and ACC (mean probability 0.71 ± 0.0066 vs 0.56 ± 0.036 pre and post lesion, *p* = 0.0041) groups. The pre-op versus post-op comparison was not significant in the PS (mean probability 0.68 ± 0.027 vs 0.69 ± 0.042 pre and post lesion, *p* = 0.37), sdlPFC (mean probability 0.83 ± 0.017 vs 0.83 ± 0.027 pre and post lesion, *p* = 0.92), and FPC (mean probability 0.68 ± 0.027 vs 0.69 ± 0.042 pre and post lesion, *p* = 0.57) groups. OFC- and ACC-lesioned animals were therefore less likely to repeat the correct choice when the value of the reinforced rule has not yet been learned and reward delivery was surprising.

**Table 7.** Full output of ANOVA on probability of repeating choice following a large positive RPE with lesion presence (two levels, pre vs post lesion) and session (10 levels, 1–10) as the within-subject variables and lesion type (five levels, ACC, PS, OFC, sdlPFC, and FPC) as the between-subject variable

	Sum of squares	df	Mean square	*F*	*p* (GG corrected)
Lesion type	4.570	4	1.143	3.269	0.046
Lesion presence	184.480	9	20.497	371.710	1.30 × 10^−18^
Session	0.224	9	0.025	1.313	0.273
Lesion type × lesion presence	6.337	36	0.176	3.192	0.014
Lesion type × session	0.619	36	0.017	0.909	0.573
Lesion presence × session	184.760	20	9.238	260.380	1.72 × 10^−26^
Lesion presence × lesion type × session	7.039	80	0.088	2.480	0.015

None of the investigated lesions significantly affected the probability of repeating choices following trials with high negative RPEs (RPE = −1 to −0.75; Fig. 5*C*). The results of a repeated-measure ANOVA (see Materials and Methods) revealed a significant interaction (*F*_(36,108)_ = 2.367, *p* = 0.007; see Table 8 for full ANOVA output) between lesion presence (pre-op vs post-op) and lesion type (ACC, PS, OFC, sdlPFC, FPC) on the probability of repeating choices following high negative RPE trials. Yet, the results of planned post hoc testing on the estimated marginal means corrected comparisons revealed that the probability of repeating choices was not significantly affected in any of the lesion groups: ACC (mean probability 0.57 ± 0.042, vs 0.65 ± 0.04 pre and post lesion, *p* = 0.14, Bonferroni’s correction for multiple comparisons), PS (mean probability 0.60 ± 0.04 vs 0.54 ± 0.056 pre and post lesion, *p* = 0.17), OFC (mean probability 0.65 ± 0.049 vs 0.60 ± 0.060 pre and post lesion, *p* = 0.60), sdlPFC (mean probability 0.43 ± 0.47 vs 0.45 ± 0.039 pre and post lesion, *p* = 0.71), and FPC (mean probability 0.40 ± 0.026 vs 0.36 ± 0.027 pre and post lesion, *p* = 0.51).

**Table 8.** Full output of ANOVA on probability of repeating choice following a large negative RPE with lesion presence (two levels, pre vs post lesion) and session (10 levels, 1–10) as the within-subject variables and lesion type (five levels, ACC, PS, OFC, sdlPFC, and FPC) as the between-subject variable

	Sum of squares	df	Mean square	*F*	*p* (GG corrected)
Lesion type	3.608	4	0.902	4.313	0.019
Lesion presence	96.938	9	10.771	160.200	9.28 × 10^−32^
Session	0.617	9	0.069	1.302	0.271
Lesion type × lesion presence	5.730	36	0.159	2.367	0.007
Lesion type × session	2.267	36	0.063	1.197	0.280
Lesion presence × session	97.544	20	4.877	73.769	1.57 × 10^−39^
Lesion presence × lesion type × session	8.537	80	0.107	1.614	0.037

Next, we analyzed choice behavior following small RPE (RPE = 0 to 0.25) to investigate lesion effects on performance when the value of the matching rules was well established. Small positive prediction errors follow reward delivery which is unsurprising, given the high expected value associated with the chosen rule (e.g., following within block correct choices). Small negative prediction errors arise when no reward is delivered following the application of a rule with low expected value, and the difference between expected and actual reward is thus small (e.g., after a within block error).

The ACC, PS, and OFC lesion groups were significantly impaired at applying the correct rule following trials with a small positive prediction error. The probability of repeating choices following small positive RPE trials can be seen in Figure 5*D*. The results of a repeated-measure ANOVA (see Materials and Methods) on the animals’ choices following small positive RPE values showed a significant main effect (*F*_(9,117)_ = 1,203.2, *p* = 1.97 × 10^−20^) of lesion presence (pre-op vs post-op) on the probability of repeating choices following small RPE trials (see Table 9 for full ANOVA output). Post hoc testing revealed a significant main effect of lesion presence in the PS (mean probability 0.83 ± 0.0060 vs 0.77 ± 0.016 pre and post lesion, *p* = 0.029) and OFC (mean probability 0.88 ± 0.0075 vs 0.70 ± 0.021 pre and post lesion, *p* = 0.00045) groups. However, the effect of lesion was not significant in the AC (mean probability 0.81 ± 0.0062 vs 0.74 ± 0.018 pre and post lesion, *p* = 0.064), sdlPFC (mean probability 0.89 ± 0.013 vs 0.89 ± 0.0094 pre and post lesion, *p* = 0.91), or FPC (mean probability 0.89 ± 0.0046 vs 0.92 ± 0.0038 pre and post lesion, *p* = 0.47) groups.

**Table 9.** Full output of ANOVA on probability of repeating choice following a small positive RPE with lesion presence (two levels, pre vs post lesion) and session (10 levels, 1–10) as the within-subject variables, and lesion type (five levels, ACC, PS, OFC, sdlPFC, and FPC) as the between-subject variable

	Sum of squares	df	Mean square	*F*	*p* (GG corrected)
Lesion type	1.073	4	0.268	1.696	0.211
Lesion presence	242.610	9	26.957	1,203.200	1.97 × 10^−20^
Session	0.065	9	0.007	2.103	0.079
Lesion type × lesion presence	1.561	36	0.043	1.936	0.125
Lesion type × session	0.148	36	0.004	1.196	0.290
Lesion presence × session	242.690	20	12.134	983.850	7.05 × 10^−27^
Lesion presence × lesion type × session	1.778	80	0.022	1.801	0.116

Finally, none of the tested frontal lesions significantly affected the probability of repeating choices following trials with small negative prediction errors (RPE = −0.25 to 0; Fig. 5*E*). A repeated-measure ANOVA (see Materials and Methods) on small negative RPE values revealed a significant interaction (*F*_(36,108)_ = 2.182, *p* = 0.012) between lesion type (ACC, PS, OFC, sdlPFC, and FPC) and lesion presence (pre-op vs post-op) on the probability of repeating choices following trials with small RPEs (see Table 10 for full ANOVA output). The results of post hoc testing on the estimated marginal means did not reveal a significant change in the probability of repeating choices for any of the lesion groups: ACC (mean probability 0.56 ± 0.0062 vs 0.47 ± 0.018 pre and post lesion, *p* = 0.18, Bonferroni’s correction for multiple comparisons), PS (mean probability 0.52 ± 0.0060 vs 0.49 ± 0.016 pre and post lesion, *p* = 0.56), OFC (mean probability 0.64 ± 0.0075 vs 0.50 ± 0.021 pre and post lesion, *p* = 0.067), sdlPFC (mean probability 0.66 ± 0.013 vs 0.62 ± 0.0094 pre and post lesion, *p* = 0.54), and FPC (mean probability 0.67 ± 0.0046 vs 0.76 ± 0.0038 pre and post lesion, *p* = 0.13).

**Table 10.** Full output of ANOVA on probability of repeating choice following a small negative RPE with lesion presence (two levels, pre vs post lesion) and session (10 levels, 1–10) as the within-subject variables and lesion type (five levels, ACC, PS, OFC, sdlPFC, and FPC) as the between-subject variable

	Sum of squares	df	Mean square	*F*	*p* (GG corrected)
Lesion type	2.480	4	0.620	5.951	0.006
Lesion presence	122.910	9	13.656	246.250	3.22 × 10^−38^
Session	0.247	9	0.027	0.668	0.612
Lesion type × lesion presence	4.356	36	0.121	2.182	0.012
Lesion type × session	1.326	36	0.037	0.895	0.575
Lesion presence × session	123.390	20	6.170	140.410	2.14 × 10^−42^
Lesion presence × lesion type × session	5.740	80	0.072	1.633	0.050

## Discussion

We have applied a three-parameter feedback-dependent RL model to behavioral data obtained from macaques with circumscribed lesions to the PS, ACC, OFC, sdlPFC, and FPC. We show that OFC lesions significantly affected the animals’ ability to use both positive and negative reinforcement to evaluate abstract matching rules and exploit the optimal rule by repeating rewarded choices. In the ACC group, animals were significantly less efficient at using negative feedback to update rule value representations. Choice behavior was also impaired following highly surprising reward delivery, consistent with the proposed role of the ACC in supporting decision-making under increased error likelihood (Kuwabara et al., 2014). In contrast to the OFC and ACC, the WCST performance impairment found in the PS group cannot be accounted for by a deficit in rule value learning; animals with PS lesions were less likely to consistently apply correctly identified rules. Therefore, by obtaining a numerical estimate of the rate of learning from positive and negative feedback, as well as relating trial-by-trial choice behavior to current reward expectations, we highlight a new approach to distinguish between component cognitive mechanisms contributing to WCST performance. Lastly, our results show that WCST performance does not necessitate intact functioning of the FPC or sdlPFC, further emphasizing the functional dissociation within the prefrontal and medial frontal regions.

Our results suggest that intact functioning of the OFC is necessary for using reward information to learn the values of abstract rules in the WCST. Learning from both reward delivery and reward omission was less efficient in the OFC group, as our FD-RL model predicted that OFC lesions were associated with a significant decrease in both positive and negative learning rate (Fig. 4*C*,*D*). OFC-lesioned animals were particularly impaired on WCST trials in which the current rule value was not well established (Figs. 2*C*, 5*B*). Following surprising reward delivery that is after a high positive prediction error, OFC-lesioned monkeys were less likely to repeat the rewarded choice than unoperated controls (Fig. 5*B*). This is consistent with previous behavioral findings showing a significant OFC lesion-related impairment on choice behavior following a single correct answer (Buckley et al., 2009). Our model further suggests that the value updating impairment in the OFC group is not limited to learning from rewards but extends to value updating in response to award omission. These results align with previous findings showing that the OFC is necessary for learning changing stimulus–value, reward–value, and action–value associations (Butter, 1969; Meunier et al., 1997; Izquierdo et al., 2004; Izquierdo and Murray, 2005). In object reversal tasks, where animals must shift to a new object after selecting a previously rewarded object that no longer produces reward, OFC-lesioned monkeys make more errors both by failing to change their response after an error and failing to stay after a correct response (Rudebeck and Murray, 2008). We extend these findings by showing that OFC lesions significantly impair the rate of learning from negative as well as positive outcomes when updating the expected value of abstract rules (as it is rules not stimuli, actions, or rewards whose values are periodically changing in the WCST analog). Additionally, the FD-RL model predicts that post-lesion performance in both the OFC and ACC group can be characterized by an increased level of noise included in the transformation of value estimates into categorical choices in favor of a single rule (Fig. 4*E*), which suggests both areas are involved in integrating past choices and outcomes to guide future rule-based decision-making.

In the ACC, we observed a deficit in value updating specifically following negative feedback; the interpretation of model-predicted ACC lesion effects is not straightforward, however (Fig. 4*C*,*D*). In contrast to the OFC lesions (impaired in both scenarios), ACC lesions were associated with a significant decrease in the best fitting values of negative, but not positive, learning rate. This finding likely reflects monkeys in the ACC group requiring more instances of reward omission to accurately estimate current rule value than unoperated controls. Despite the lack of significant lesion effect on the rate of value updating following positive feedback, we identified a significant effect of ACC lesion on the probability of repeating a rewarded rule application following trials in which the delivery of reward was surprising (high positive reward prediction error trials; Fig. 5*A*). This is consistent with previous behavioral findings showing a significant ACC lesion-related decrease in the probability of correct choice on trials preceded by a single correct choice in the WCST (Buckley et al., 2009). It therefore cannot be said that the ACC impairment in the WCST is limited strictly to error-driven learning (Carter et al., 1998; Rushworth et al., 2004, 2007a; Mansouri et al., 2017a), as monkeys in the ACC group also exhibit impairments in adapting choice behavior to incoming positive feedback (Fig. 5*A*). Similarly, in action reversal tasks, ACC-lesioned monkeys showed evidence of decreased influence of previous reward history on action choices, as they are slower to learn from both negative and positive outcomes (Kennerley et al., 2006). This can be contrasted with lesions to the OFC, which did not produce an impairment on tasks in which new values of actions, rather than objects, must be learned (Rudebeck et al., 2008). As the OFC has more access to highly processed visual information via its connections with the temporal and perirhinal cortex than the ACC (Van Hoesen et al., 1993; Carmichael and Price, 1994; Webster et al., 1994; Kondo et al., 2005), which is in contrast more positioned to influence action selection via its direct connections to the premotor and motor cortex (Dum and Srick, 1993; Wang et al., 2004), it has been proposed that the OFC supports stimulus-based decision-making, whereas the ACC supports action-based decision-making (Rudebeck et al., 2008). Nevertheless, the ACC and OFC also interact in various ways to support a range of cognitive functions (Rushworth et al., 2007b). Here, we show that the heavily interconnected prefrontal and medial frontal cortical areas likely work together to implement decision-making based on higher-order constructs such as abstract rules: in the WCST, where decisions are guided by evaluating abstract matching rules, both the OFC and ACC likely contribute to integrating past choices and their outcomes to guide rule-based actions.

The ACC lesion group deficits we observed can be explained in light of a recent proposal that WCST performance relies on multiple modes of response selection. It has been suggested that WCST performance relies on two modes of response selection, whereby samples are chosen based on the working memory representation of the current rule when rule values are well established, and a trial-and-error mode relying on rule value estimates is engaged when high error likelihood or uncertainty is detected (Kuwabara et al., 2014). The ACC may be particularly important for selecting and switching between these two response modes based on the current levels of error likelihood or uncertainty. This is distinct from switching between the color and shape matching rules following a change in rule–reward contingency. In fact, ACC lesions do not impair rule-switching per se, as there was no lesion effect on the number of errors made on the first trial after an unannounced rule change (Fig. 2*A*). Rather, there is evidence to show that the ACC supports flexible switching between working memory-based and trial-and-error–based response modes to flexibly adjust decision-making strategy to the current levels of uncertainty. Single-cell recordings obtained from the ACC while monkeys were performing the WCST suggest that ACC represents both context-dependent and internally detected error likelihoods (Kuwabara et al., 2014). In a decision-making task with explicitly cued exploration and exploitation stages, ACC supports behavioral shifts between exploration and exploitation by signaling first instances of reward in the exploration stage (Quilodran et al., 2008). The WCST performance deficits we observed in the ACC group are consistent with ACC-lesioned monkeys remaining longer in the trial-and-error response mode, requiring relatively more trials to engage in choice selection driven by WM rule representations following the detection of increased reward uncertainty. The lesion-related decrease in negative learning rate suggests value updating is less efficient following error trials, associated with increased error likelihood. Similarly, the deficit in repetition of rewarded choice following large but not small positive RPEs reflects an impairment in adapting to surprising changes in rule–reward contingency.

Our results show that learning the value of the currently reinforced rule in the WCST does not require intact functioning of the PS. As we found no lesion-related difference in any of the model parameters, the performance impairment in the WCST observed in the PS group cannot be explained by a deficit in feedback-driven rule value updating or choice behavior. Monkeys in the PS group were less likely to repeat a rewarded choice on trials in which the value of the rule had already been well learned (trials following a small positive reward prediction error; Fig. 5*D*). Post-lesion performance was therefore impaired particularly on trials in which choice behavior relies primarily on the continued application of the currently relevant rule. To successfully perform the WCST, subjects must maintain in WM the identity of a previously chosen matching rule (not just the specific stimulus features as in other WCST analog, e.g., Goudar et al., 2024), to determine whether to match samples based on their shape or color across successive presentations of samples with different individual features. Single-cell recording revealed that neurons in the dorsolateral prefrontal cortex (dlPFC) encode the currently relevant rule in a WCST analog, as cell activity was modulated by rule type in each block, regardless of individual sample identity (Mansouri et al., 2006), suggesting that the dlPFC may play a crucial role in the representation of rule identity. Bidirectional connections between the dorsolateral and orbitofrontal cortices possibly support the updating of rule representations to reflect current rule value in light of recent choice feedback. Lesions to the principal sulcus within the inferior dlPFC, but not lesions to the superior dlPFC, render monkeys particularly vulnerable to taxing WM by increasing the length of the intertrial interval in the WCST (Buckley et al., 2009). Taken together, our results therefore support the idea that the PS is necessary for maintaining the representation of abstract rules in WM to guide choice behavior when the optimal rule has been identified. Future research may test this idea further by adding a WM component to the reinforcement learning model, which by default does not formalize the effect of WM capacity and duration on choice behavior (Collins and Frank, 2012).

In contrast to the ACC, OFC, and PS, our results further confirm that it is possible to perform the WCST analog without the involvement of sdlPFC and FPC (Buckley et al., 2009; Mansouri et al., 2015), which further highlights the functional dissociation between prefrontal regions in rule-guided decision-making. Consistently, we detected no model-predicted differences in the process by which animals with sdlPFC and FPC lesions learn the value of the two abstract rules based on incoming feedback and make categorical decisions in favor of one rule. This is not to say the FPC is not involved in the WCST in all circumstances, for example, when the WM for rule is taxed by adding distractors in the intertrial interval, FPC-lesioned animals suffer less from distraction and outperform unoperated control animals (Mansouri et al., 2015). It has been proposed that the FPC is involved in the redistribution of cognitive resources away from the current task to explore new opportunities (Mansouri et al., 2015, 2017b). Therefore, the aforementioned lesion effect in WCST could be advantageous in such circumstances by preventing exploration of the relative value of deleterious distractors. Consistent with this hypothesis, a recent study has reported that electrical microstimulation of FPC modified WCST performance by decreasing exploration and impairing adaptation to rule changes. Crucially in this study both animals developed a behavioral strategy in which they attached value to, and periodically explored, the alternative rule to pre-empt block changes (Ainsworth et al., 2024).

The evidence of functional specialization within the frontal cortex presented here refutes some early ideas about functional architecture of the broad region, including that the prefrontal cortex is functionally undifferentiated, that a simple hierarchy exists as one proceeds anteriorly, or that there are dorsal versus ventral spatial versus nonspatial domain specific subdivisions (for reviews of earlier proposals, see Rushworth, 2000; Curtis and D’esposito, 2004; Szczepanski and Knight, 2014). Instead, we show evidence of a dissociation between lesions to the ACC and OFC, impairing the integration of choice feedback to guide rule-based decision-making, and lesions to the PS affecting the maintenance of abstract rule representations in WM. The ventrolateral prefrontal cortex (vlPFC) is a likely candidate for a domain general hub for maintaining the basic task structure (relations between actions stimuli, rules, strategies, and goals; Kadohisa et al., 2023), as lesions to the inferior convexity of vlPFC were the only frontal lesions that rendered animals incapable of performing the WCST analog, exhibiting significant impairments at a control task assessing rule-guided matching abilities without rule shifting (Buckley et al., 2009). It has been suggested that orchestrated interactions between these bidirectionally interconnected regions act to maximize exploitation of the current goal-directed behavior (Mansouri et al., 2017b). In volatile environments, where animals also need to flexibly control the relative amount of exploration of alternatives to the current goal, we have proposed FPC as a candidate region contributing to such exploration (Mansouri et al., 2015, 2017b; Boschin et al., 2025).

In summary, our results obtained by combining lesioning with reinforcement learning modeling further highlight the dissociable contributions of distinct prefrontal and medial frontal regions to complex rule-based decision-making. We identified a key distinction between a rule value updating component of the task, which necessitates intact functioning of the ACC and OFC, and a working memory component, which primarily relies on the PS. We further suggest that the ACC may have a critical role in switching between these two response modes in the WCST. Our analyses highlight how the distinct components of WCST performance can be disambiguated by examining lesion effects on RL model-predicted parameters capturing distinct latent cognitive mechanisms. Results of lesion studies, informing us what contribution each area is necessary for, therefore generate location-specific hypotheses for future work with multiarea multielectrode recordings which can advance our understanding of the mechanisms and temporal dynamics of network-level interactions underlying rule-guided decision-making.

## Synthesis

Reviewing Editor: Mark Laubach, American University Decisions are customarily a result of the Reviewing Editor and the peer reviewers coming together and discussing their recommendations until a consensus is reached. When revisions are invited, a fact-based synthesis statement explaining their decision and outlining what is needed to prepare a revision will be listed below. The following reviewer(s) agreed to reveal their identity: Mark Eldridge. Note: If this manuscript was transferred from JNeurosci and a decision was made to accept the manuscript without peer review, a brief statement to this effect will instead be what is listed below.

Your paper was reviewed by a previous reviewer and they asked for a few minor changes to be made. Their comments are below. Please revise accordingly and the paper will be reviewed editorially after we receive your revisions.

The authors have performed comprehensive additional analyses and have expanded the contextualization and interpretation of the study to emphasize the relevance of their findings.

I'm still a little uneasy that their preferred model fit is so much poorer in ACC, PS, and OFC post-lesion than it is in sdlPFC or FPC, (or in any of the pre-lesion groups) but, as the authors state, the fits are still good, and I am convinced by the relative differences among the groups post-lesion.

For transparency, it would be better if the authors included the post-lesion model fit data plot (that they provided in the rebuttal) as part of fig. 3.

All of my concerns have been satisfied in this revision.

## Author Response

Dear Prof. Laubach, Thank you for considering our manuscript for publication. We would like to thank you and the reviewers for your time and effort.

Please see attached an updated copy of the manuscript, in which we now include the post-lesion model fit data plot as a part of Figure 3 as requested by the reviewer, referenced in line 611-12 of the Article file: "Furthermore, the FD-RL model also provided the best model fit for post-lesion data out of the four model variants, as can be seen in Figure 3B." We also include the updated Clean copy with title page and individual figure files in TIFF format (at 300DPI). We believe these should meet the journal requirements, please let us know if not.

Kind Regards Lucie Capkova

## References

[B2] Ainsworth M, Wu Z, Browncross H, Mitchell AS, Bell AH, Buckley MJ (2022) Frontopolar cortex shapes brain network structure across prefrontal and posterior cingulate cortex. Prog Neurobiol 217:102314. 10.1016/j.pneurobio.2022.102314 35798212

[B1] Ainsworth M, Galeazzi JM, Pedreira C, Stokes MG, Buckley MJ (2024) Frontopolar cortex stimulation induces prolonged disruption to counterfactual processing: insights from altered local field potentials | bioRxiv. Available at: https://www.biorxiv.org/content/10.1101/2024.11.26.625398v1.full.pdf+html 10.1371/journal.pbio.3003495 PMC12674556 41252455

[B5] Boschin EA, Buckley MJ (2015) Differential contributions of dorsolateral and frontopolar cortices to working memory processes in the primate. Front Syst Neurosci 9:144. 10.3389/fnsys.2015.00144 26578901 PMC4624853

[B7] Boschin EA, Piekema C, Buckley MJ (2015) Essential functions of primate frontopolar cortex in cognition. Proc Natl Acad Sci 112:E1020–E1027. 10.1073/pnas.1419649112 25691741 PMC4352768

[B4] Boschin EA, Brkic MM, Simons JS, Buckley MJ (2017a) Distinct roles for the anterior cingulate and dorsolateral prefrontal cortices during conflict between abstract rules. Cereb Cortex 27:34–45. 10.1093/cercor/bhw350 28365775 PMC5939207

[B6] Boschin EA, Mars RB, Buckley MJ (2017b) Transcranial magnetic stimulation to dorsolateral prefrontal cortex affects conflict-induced behavioural adaptation in a Wisconsin Card Sorting Test analogue. Neuropsychologia 94:36–43. 10.1016/j.neuropsychologia.2016.11.015 27889392 PMC5226064

[B3] Boschin EA, Ainsworth M, Galeazzi JM, Buckley MJ (2025) Memories or decisions? Bridging accounts of frontopolar function. Neuropsychologia 211:109119. 10.1016/j.neuropsychologia.2025.109119 40058578

[B8] Buckley MJ, Mansouri FA, Hoda H, Mahboubi M, Browning PGF, Kwok SC, Phillips A, Tanaka K (2009) Dissociable components of rule-guided behavior depend on distinct medial and prefrontal regions. Science 325:52–58. 10.1126/science.1172377 19574382

[B9] Butter CM (1969) Perseveration in extinction and in discrimination reversal tasks following selective frontal ablations in *Macaca mulatta*. Physiol Behav 4:163–171. 10.1016/0031-9384(69)90075-4

[B10] Carmichael ST, Price JL (1994) Architectonic subdivision of the orbital and medial prefrontal cortex in the macaque monkey. J Comp Neurol 346:366–402. 10.1002/cne.903460305 7527805

[B11] Carter CS, Braver TS, Barch DM, Botvinick MM, Noll D, Cohen JD (1998) Anterior cingulate cortex, error detection, and the online monitoring of performance. Science 280:747–749. 10.1126/science.280.5364.747 9563953

[B12] Chau BKH, Sallet J, Papageorgiou GK, Noonan MP, Bell AH, Walton ME, Rushworth MFS (2015) Contrasting roles for orbitofrontal cortex and amygdala in credit assignment and learning in macaques. Neuron 87:1106–1118. 10.1016/j.neuron.2015.08.018 26335649 PMC4562909

[B13] Ciranka S, Linde-Domingo J, Padezhki I, Wicharz C, Wu CM, Spitzer B (2022) Asymmetric reinforcement learning facilitates human inference of transitive relations. Nat Hum Behav 6:555–564. 10.1038/s41562-021-01263-w 35102348 PMC9038534

[B14] Collins AGE, Frank MJ (2012) How much of reinforcement learning is working memory, not reinforcement learning? A behavioral, computational, and neurogenetic analysis. Eur J Neurosci 35:1024–1035. 10.1111/j.1460-9568.2011.07980.x 22487033 PMC3390186

[B15] Costa VD, Dal Monte O, Lucas DR, Murray EA, Averbeck BB (2016) Amygdala and ventral striatum make distinct contributions to reinforcement learning. Neuron 92:505–517. 10.1016/j.neuron.2016.09.025 27720488 PMC5074688

[B16] Curtis CE, D’esposito M (2004) The effects of prefrontal lesions on working memory performance and theory. Cogn Affect Behav Neurosci 4:528–539. 10.3758/cabn.4.4.528 15849895

[B17] De Schotten MT, Dell’Acqua F, Valabregue R, Catani M (2012) Monkey to human comparative anatomy of the frontal lobe association tracts. Cortex 48:82–96. 10.1016/j.cortex.2011.10.001 22088488

[B18] Dias R, Robbins TW, Roberts AC (1997) Dissociable forms of inhibitory control within prefrontal cortex with an analog of the Wisconsin Card Sort Test: restriction to novel situations and independence from “on-line” processing. J Neurosci 17:9285–9297. 10.1523/JNEUROSCI.17-23-09285.1997 9364074 PMC6573594

[B19] Drewe EA (1974) The effect of type and area of brain lesion on Wisconsin Card Sorting Test performance. Cortex 10:159–170. 10.1016/S0010-9452(74)80006-7 4844468

[B20] Dum R, Srick P (1993) Cingulate motor areas. In: *Neurobiology of the cingulate and limbic thalamus* (Vogt B, ed), pp 415–441. Boston: Birkhauser.

[B21] Fehring DJ, Pascoe AJ, Haque ZZ, Samandra R, Yokoo S, Abe H, Rosa MGP, Tanaka K, Yamamori T, Mansouri FA (2022) Dimension of visual information interacts with working memory in monkeys and humans. Sci Rep 12:5335. 10.1038/s41598-022-09367-7 35351948 PMC8964748

[B22] Goudar V, et al. (2024) A comparison of rapid rule-learning strategies in humans and monkeys. J Neurosci 44:e0231232024. 10.1523/JNEUROSCI.0231-23.2024 38871463 PMC11236592

[B23] Izquierdo A, Murray EA (2005) Opposing effects of amygdala and orbital prefrontal cortex lesions on the extinction of instrumental responding in macaque monkeys. Eur J Neurosci 22:2341–2346. 10.1111/j.1460-9568.2005.04434.x 16262672

[B24] Izquierdo A, Suda RK, Murray EA (2004) Bilateral orbital prefrontal cortex lesions in rhesus monkeys disrupt choices guided by both reward value and reward contingency. J Neurosci 24:7540–7548. 10.1523/JNEUROSCI.1921-04.2004 15329401 PMC6729636

[B25] Kadohisa M, Kusunoki M, Mitchell DJ, Bhatia C, Buckley MJ, Duncan J (2023) Frontal and temporal coding dynamics in successive steps of complex behavior. Neuron 111:430–443.e3. 10.1016/j.neuron.2022.11.004 36473483

[B26] Kennerley SW, Walton ME, Behrens TEJ, Buckley MJ, Rushworth MFS (2006) Optimal decision making and the anterior cingulate cortex. Nat Neurosci 9:940–947. 10.1038/nn1724 16783368

[B27] Kondo H, Saleem KS, Price JL (2005) Differential connections of the perirhinal and parahippocampal cortex with the orbital and medial prefrontal networks in macaque monkeys. J Comp Neurol 493:479–509. 10.1002/cne.20796 16304624

[B28] Kuwabara M, Mansouri FA, Buckley MJ, Tanaka K (2014) Cognitive control functions of anterior cingulate cortex in macaque monkeys performing a Wisconsin Card Sorting Test analog. J Neurosci 34:7531–7547. 10.1523/JNEUROSCI.3405-13.2014 24872558 PMC4035517

[B29] Kwok SC, Cai Y, Buckley MJ (2019) Mnemonic introspection in macaques is dependent on superior dorsolateral prefrontal cortex but not orbitofrontal cortex. J Neurosci 39:5922–5934. 10.1523/JNEUROSCI.0330-19.2019 31123101 PMC6650985

[B30] Li J, Schiller D, Schoenbaum G, Phelps EA, Daw ND (2011) Differential roles of human striatum and amygdala in associative learning. Nat Neurosci 14:1250–1252. 10.1038/nn.2904 21909088 PMC3268261

[B31] Mahmoodi A, Harbison C, Bongioanni A, Emberton A, Roumazeilles L, Sallet J, Khalighinejad N, Rushworth MFS (2024) A frontopolar-temporal circuit determines the impact of social information in macaque decision making. Neuron 112:84–92.e6. 10.1016/j.neuron.2023.09.035 37863039 PMC10914637

[B43] Mansouri FA, Tanaka K (2002) Behavioral evidence for working memory of sensory dimension in macaque monkeys. Behav Brain Res 136:415–426. 10.1016/S0166-4328(02)00182-1 12429403

[B42] Mansouri FA, Matsumoto K, Tanaka K (2006) Prefrontal cell activities related to monkeys’ success and failure in adapting to rule changes in a Wisconsin Card Sorting Test analog. J Neurosci 26:2745–2756. 10.1523/JNEUROSCI.5238-05.2006 16525054 PMC6675148

[B34] Mansouri FA, Buckley MJ, Tanaka K (2007) Mnemonic function of the dorsolateral prefrontal cortex in conflict-induced behavioral adjustment. Science 318:987–990. 10.1126/science.1146384 17962523

[B44] Mansouri FA, Tanaka K, Buckley MJ (2009) Conflict-induced behavioural adjustment: a clue to the executive functions of the prefrontal cortex. Nat Rev Neurosci 10:141–152. 10.1038/nrn2538 19153577

[B35] Mansouri FA, Buckley MJ, Tanaka K (2014) The essential role of primate orbitofrontal cortex in conflict-induced executive control adjustment. J Neurosci 34:11016–11031. 10.1523/JNEUROSCI.1637-14.2014 25122901 PMC4131015

[B33] Mansouri FA, Buckley MJ, Mahboubi M, Tanaka K (2015) Behavioral consequences of selective damage to frontal pole and posterior cingulate cortices. Proc Natl Acad Sci 112:E3940–E3949. 10.1073/pnas.1422629112 26150522 PMC4517212

[B39] Mansouri FA, Fehring DJ, Feizpour A, Gaillard A, Rosa MGP, Rajan R, Jaberzadeh S (2016) Direct current stimulation of prefrontal cortex modulates error-induced behavioral adjustments. Eur J Neurosci 44:1856–1869. 10.1111/ejn.13281 27207192

[B38] Mansouri FA, Egner T, Buckley MJ (2017a) Monitoring demands for executive control: shared functions between human and nonhuman primates. Trends Neurosci 40:15–27. 10.1016/j.tins.2016.11.001 27986294

[B41] Mansouri FA, Koechlin E, Rosa MGP, Buckley MJ (2017b) Managing competing goals — a key role for the frontopolar cortex. Nat Rev Neurosci 18:645–657. 10.1038/nrn.2017.111 28951610

[B32] Mansouri FA, Buckley MJ, Fehring DJ, Tanaka K (2020a) The role of primate prefrontal cortex in bias and shift between visual dimensions. Cereb Cortex 30:85–99. 10.1093/cercor/bhz072 31220222 PMC7029686

[B40] Mansouri FA, Freedman DJ, Buckley MJ (2020b) Emergence of abstract rules in the primate brain. Nat Rev Neurosci 21:595–610. 10.1038/s41583-020-0364-5 32929262

[B36] Mansouri FA, Buckley MJ, Tanaka K (2022) The neural substrate and underlying mechanisms of executive control fluctuations in primates. Prog Neurobiol 209:102216. 10.1016/j.pneurobio.2022.102216 34995695

[B37] Mansouri FA, Buckley MJ, Tanaka K (2024) Mapping causal links between prefrontal cortical regions and intra-individual behavioral variability. Nat Commun 15:140. 10.1038/s41467-023-44341-5 38168052 PMC10762061

[B45] McFadden D (1974) Conditional logit analysis of qualitative choice behaviour. In: *Frontiers in economics* (Zarembka P, ed), pp 105–142. New York: Academic Press.

[B46] Meunier M, Bachevalier J, Mishkin M (1997) Effects of orbital frontal and anterior cingulate lesions on object and spatial memory in rhesus monkeys. Neuropsychologia 35:999–1015. 10.1016/S0028-3932(97)00027-4 9226661

[B47] Milner B (1963) Effects of different brain lesions on card sorting: the role of the frontal lobes. Arch Neurol 9:90–100. 10.1001/archneur.1963.00460070100010

[B48] Moore TL, Schettler SP, Killiany RJ, Rosene DL, Moss MB (2009) Effects on executive function following damage to the prefrontal cortex in the rhesus monkey (*Macaca mulatta*). Behav Neurosci 123:231–241. 10.1037/a0014723 19331446

[B50] Nakahara K, Hayashi T, Konishi S, Miyashita Y (2002) Functional MRI of macaque monkeys performing a cognitive set-shifting task. Science 295:1532–1536. 10.1126/science.1067653 11859197

[B49] Nakahara K, Adachi Y, Osada T, Miyashita Y (2007) Exploring the neural basis of cognition: multi-modal links between human fMRI and macaque neurophysiology. Trends Cogn Sci 11:84–92. 10.1016/j.tics.2006.11.006 17188927

[B51] Nelson HE (1976) A modified card sorting test sensitive to frontal lobe defects. Cortex 12:313–324. 10.1016/S0010-9452(76)80035-4 1009768

[B52] Petrides M, Pandya DN (1994) Comparative architectonic analysis of the human and macaque frontal cortex. In: *Handbook of neuropsychology* (Boller F, Grafman J, eds), pp 17–58. Amsterdam: Elsevier.

[B53] Petrides M, Pandya DN (1999) Dorsolateral prefrontal cortex: comparative cytoarchitectonic analysis in the human and the macaque brain and corticocortical connection patterns. Eur J Neurosci 11:1011–1036. 10.1046/j.1460-9568.1999.00518.x 10103094

[B54] Quilodran R, Rothé M, Procyk E (2008) Behavioral shifts and action valuation in the anterior cingulate cortex. Neuron 57:314–325. 10.1016/j.neuron.2007.11.031 18215627

[B55] Rudebeck PH, Behrens TE, Kennerley SW, Baxter MG, Buckley MJ, Walton ME, Rushworth MFS (2008) Frontal cortex subregions play distinct roles in choices between actions and stimuli. J Neurosci 28:13775–13785. 10.1523/JNEUROSCI.3541-08.2008 19091968 PMC6671924

[B56] Rudebeck PH, Murray EA (2008) Amygdala and orbitofrontal cortex lesions differentially influence choices during object reversal learning. J Neurosci 28:8338–8343. 10.1523/JNEUROSCI.2272-08.2008 18701696 PMC2556079

[B57] Rudebeck PH, Saunders RC, Prescott AT, Chau LS, Murray EA (2013) Prefrontal mechanisms of behavioral flexibility, emotion regulation and value updating. Nat Neurosci 16:1140–1145. 10.1038/nn.3440 23792944 PMC3733248

[B58] Rushworth MFS (2000) Anatomical and functional subdivision within the primate lateral prefrontal cortex. Psychobiology 28:187–196. 10.3758/BF03331977

[B61] Rushworth MFS, Walton ME, Kennerley SW, Bannerman DM (2004) Action sets and decisions in the medial frontal cortex. Trends Cogn Sci 8:410–417. 10.1016/j.tics.2004.07.009 15350242

[B60] Rushworth MFS, Buckley MJ, Behrens TE, Walton ME, Bannerman DM (2007a) Functional organization of the medial frontal cortex. Curr Opin Neurobiol 17:220–227. 10.1016/j.conb.2007.03.001 17350820

[B59] Rushworth MFS, Behrens TEJ, Rudebeck PH, Walton ME (2007b) Contrasting roles for cingulate and orbitofrontal cortex in decisions and social behaviour. Trends Cogn Sci 11:168–176. 10.1016/j.tics.2007.01.004 17337237

[B62] Schmahmann J, Pandya D (2006) Fiber pathways of the brain. New York: Oxford University Press.

[B63] Sleezer BJ, Castagno MD, Hayden BY (2016) Rule encoding in orbitofrontal cortex and striatum guides selection. J Neurosci 36:11223–11237. 10.1523/JNEUROSCI.1766-16.2016 27807165 PMC5148240

[B64] Steinke A, Lange F, Kopp B (2020) Parallel model-based and model-free reinforcement learning for card sorting performance. Sci Rep 10:15464. 10.1038/s41598-020-72407-7 32963297 PMC7508815

[B65] Sutton RS, Barto AG (1998) Reinforcement learning: an introduction. IEEE Trans Neural Netw 9:1054–1054. 10.1109/TNN.1998.712192

[B66] Szczepanski SM, Knight RT (2014) Insights into human behavior from lesions to the prefrontal cortex. Neuron 83:1002–1018. 10.1016/j.neuron.2014.08.011 25175878 PMC4156912

[B67] Tanaka K, Matsumoto K, Mansouri F, Buckley M (2013) Functional division among monkey prefrontal areas in goal-directed behaviour. In: *Principles of frontal lobe function* (Stuss DT, Knight RT, eds), pp 249–258. New York: Oxford University Press.

[B68] Van Hoesen G, Morecraft B, Vogt B (1993) Connections of the monkey cingulate cortex. In: *Neurobiology of the cingulate cortex and limbic thalamus* (Vogt B, Gabriel M, eds), pp 249–284. Boston: Birkhauser.

[B69] Wang Y, Matsuzaka Y, Shima K, Tanji J (2004) Cingulate cortical cells projecting to monkey frontal eye field and primary motor cortex. Neuroreport 15:1559–1563. 10.1097/01.wnr.0000133300.62031.9b 15232283

[B70] Webster MJ, Bachevalier J, Ungerleider LG (1994) Connections of inferior temporal areas TEO and TE with parietal and frontal cortex in macaque monkeys. Cereb Cortex 4:470–483. 10.1093/cercor/4.5.470 7530521

