# Supplementary figures and images for: Dissociating Frontal Lobe Lesion Induced Deficits in Rule Value Learning Using Reinforcement Learning Models and a WCST Analog
Source: eNeuro. 2025 May 16;12(5):ENEURO.0117-25.2025. doi: 10.1523/ENEURO.0117-25.2025 (PMC12184873; doi:10.1523/ENEURO.0117-25.2025)

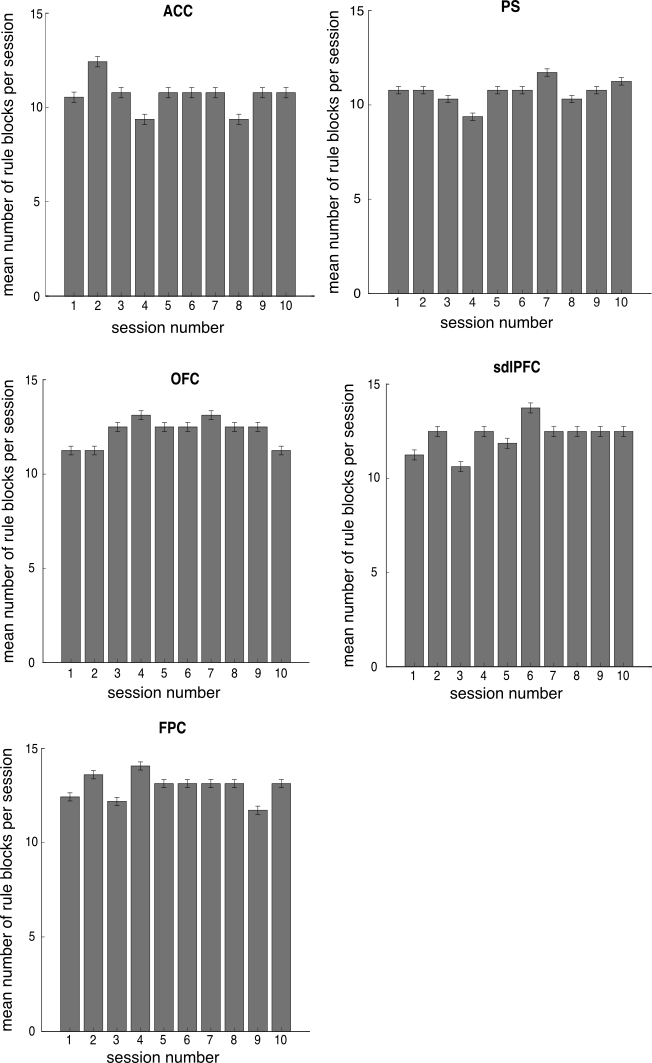

Supplement: Figure 1-1 — Bar chart showing the pre-operative number of rule blocks achieved per session, averaged across all monkeys in the ACC, PS, OFC, sdlPFC and FPC lesion groups. Error bars represent SEM. Download Figure 1-1, TIF file. [file eneuro-12-ENEURO.0117-25.2025-s001.tif]

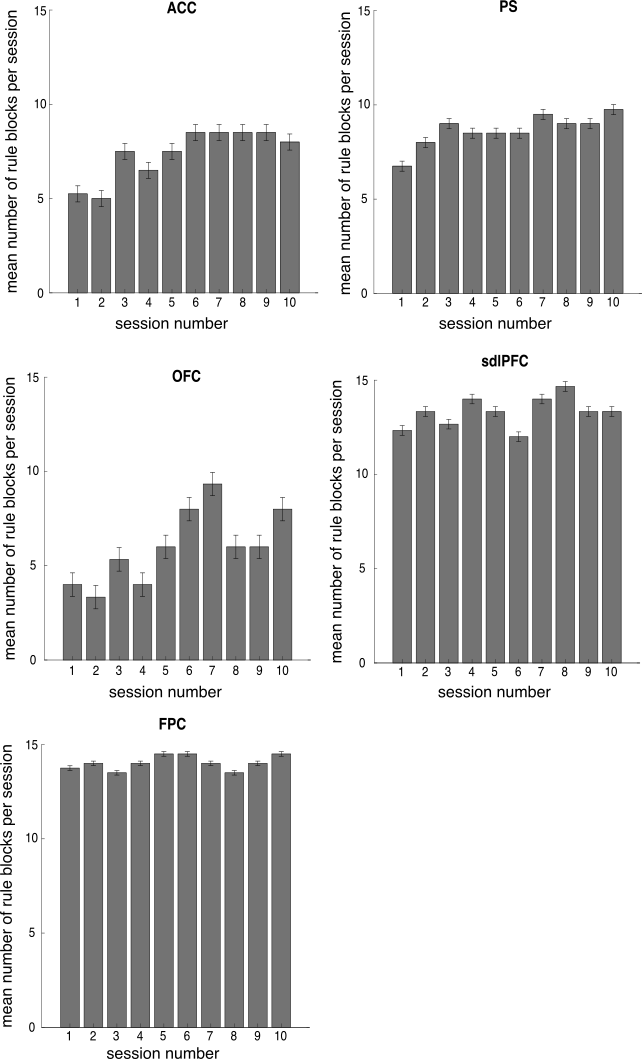

Supplement: Figure 1-2 — Bar chart showing the post-operative number of rule blocks achieved per session, averaged across all monkeys in the ACC, PS, OFC, sdlPFC and FPC lesion groups. Error bars represent SEM. Download Figure 1-2, TIF file. [file eneuro-12-ENEURO.0117-25.2025-s002.tif]
